# Overexpression of SH2D1A promotes cancer progression and is associated with immune cell infiltration in hepatocellular carcinoma via bioinformatics and in vitro study

**DOI:** 10.1186/s12885-023-11315-1

**Published:** 2023-10-19

**Authors:** Qian-Ming Xiang, Ni Jiang, Yue-Feng Liu, Yuan-Biao Wang, De-An Mu, Rong Liu, Lu-Yun Sun, Wei Zhang, Qiang Guo, Kai Li

**Affiliations:** 1 Department of Cardiothoracic surgery, Taihe Hospital, Hubei University of Medicine, Shiyan, China; 2 Department of General Surgery, The First Affiliated Hospital of Kunming Medical University, Kunming, China; 3 Department of Obstetrics and Gynecology, Women and Children’s Hospital of Chongqing Medical University, Chongqing, China; 4 Department of Ophthalmology surgery, Taihe Hospital, Hubei University of Medicine, Shiyan, China; 5 Department of Yunnan Tumor Research Institute, The Third Affiliated Hospital of Kunming Medical University, Kunming, China; 6 Department of Hepatobiliary and Pancreatic Surgery, The People’s Hospital of Jianyang city, Jianyang, China

**Keywords:** SH2 domain containing 1A, Hepatocellular carcinoma, Immune microenvironment, Prognosis, The Cancer Genome Atlas

## Abstract

**Background:**

SH2 domain containing 1A **(**SH2D1A) expression has been linked to cancer progression. However, the functions of SH2D1A in hepatocellular carcinoma (HCC) have not been reported.

**Methods:**

The effects of SH2D1A on the proliferation, migration, and invasion of HCC cells and the related pathways were re-explored in cell models with SH2D1A overexpression using the CCK-8, migration and invasion assays and western blotting. The functions and mechanisms of genes co-expressed with SH2D1A were analyzed using gene ontology (GO) and Kyoto Encyclopedia of Genes and Genomes (KEGG) analysis. The relationship between SH2D1A expression and immune microenvironment features in HCC was explored.

**Results:**

Elevated SH2D1A expression promoted cell proliferation, migration, and invasion, which was related to the overexpression of p-Nf-κB and BCL2A1 protein levels in HCC. SH2D1A expression was related to the immune, stromal, and ESTIMATE scores, and the abundance of immune cells, such as B cells, CD8^+^ T cells, and T cells. SH2D1A expression was significantly related to the expression of immune cell markers, such as PDCD1, CD8A, and CTLA4 in HCC.

**Conclusion:**

SH2D1A overexpression was found to promote cell growth and metastasis via the Nf-κB signaling pathway and may be related to the immune microenvironment in HCC. The findings indicate that SH2D1A can function as a biomarker in HCC.

**Supplementary Information:**

The online version contains supplementary material available at 10.1186/s12885-023-11315-1.

## Introduction

In recent years, hepatocellular carcinoma (HCC) has been identified as one of the most common digestive tract tumors. At present, the prognosis of patients with HCC remains nonideal. Some biomarkers can predict the prognosis of HCC patients and have been shown to promote or inhibit the growth and migration of HCC cells [1–4]. For example, transmembrane protein 166 (TMEM166) is downregulated and is related to the TNM stage and dismal prognosis in HCC. TMEM166 overexpression suppresses cancer cell growth and metastasis potential by upregulating the anticancer gene TP53. TP53 downregulation can alleviate the antitumor effects of TMEM166 on HCC cells [1]. HMGA1 is overexpressed, and its expression is related to the Edmondson grade and overall survival (OS) in HCC. Downregulation of high-mobility group protein A1 (HMGA1) significantly reduces the cell growth and metastasis potential of HCC cells. The downregulation of miR-195-5p abrogates the effects of HMGA1 on HCC cells [4]. Therefore, it is crucial to identify novel biomarkers to predict the survival time of patients with HCC, and inhibit or promote the expression of biomarkers to delay HCC progression.

The Cancer Genome Atlas (TCGA) database is often used to identify new cancer biomarkers [2, 5–7]. For example, patients with HCC with mitochondrial fission regulator 2 (MTFR2) overexpression have poorer OS, which is associated with cancer stage, age, T stage, and grade. MTFR2 overexpression can independently predict a dismal prognosis in HCC [2]. Findings from research confirm that SH2 domain containing 1A (SH2D1A) encodes a protein that plays a critical role in the bidirectional stimulation of immune cells. The SH2D1A-encoded protein binds to lymphocyte-activating signaling molecules to inhibit the expression of transmembrane proteins and surface molecules on activated B, T, and NK cells, thus altering signal transduction in the cells. Thus far, the relationship between SH2D1A and HCC has not been reported in the literature. Therefore, the roles of SH2D1A and the involved pathways were explored via CCK-8 assay, Transwell assay, Kyoto Encyclopedia of Genes and Genomes (KEGG) enrichment analysis, and western blotting, and the relationship between the immune microenvironment and SH2D1A expression was explored to evaluate the value of SH2D1A as a biomarker for HCC.

## Materials and methods

### Cell culture and models

Human normal liver cells (LO2) and HCC cells (LM3, Hep3B, PLC, HepG2, and Huh7) were purchased from The Cell Bank of the Typical Culture Preservation Committee of the Chinese Academy of Sciences, placed in an incubator at 37 °C and 5% CO_2_ and cultured in 10% fetal bovine serum (FBS) culture and 1% penicillin‒streptomycin DMEM. The SH2D1A overexpression vector (SH2D1A-OV) and negative control (Control) were synthesized by Shanghai Gemma Gene Pharmaceutical Co., Ltd. (China). LM3 and HepG2 cells were inoculated in 6-well plates and transfected after the cell confluence reached 50%. Transfection was performed according to the manufacturer's instructions using Lipofectamine 2000 transfection reagent. The solution was replaced at 6 h after transfection, and HCC cells were collected at 48 h after transfection.

### Identification of HCC cell models using RT‒PCR and Western blotting

After cell transfection, the total RNA and protein from normal liver cells and HCC cells were extracted using TRIzol and lysis buffer. After total RNA quantification, SH2D1A mRNA expression in normal and HCC cells was determined using reverse transcription and PCR amplification. The following primers were used: SH2D1A forward: 5'-CATGGCAAAATCAGCAGGAAACC-3'; reverse: 5'- AACCGTGATACAGCACACATAGGC-3'; GAPDH forward: 5′- GGAGTCCACTG GCGTCTTCA-3′; reverse: 5′-GTCATGAGTCCTTCCACGATACC-3′. Total protein was quantified using the BCA method. Twenty micrograms of the extracted protein were subjected to protein gel electrophoresis, and the membranes were treated with SH2D1A, p-Nf-κB, Nf-κB, BCL2A1, and GAPDH antibodies at the 1:1000 dilution after the membranes of western blotting were cut. Then the membranes were incubated with a secondary antibody at the 1:10,000 dilution, washed and exposed to detect SH2D1A protein expression in normal liver and HCC cells.

### Cell proliferation assay

LM3 and HepG2 cell suspensions were prepared and inoculated into 96-well plates at 2 × 10^3^ cells/well. Ten microliters of CCK-8 solution was added to 96-well plates, and the absorbance value of the cells after incubation for 2 h was measured to generate a cell proliferation curve.

### Cell migration and invasion assays

HCC cells were resuspended in serum-free medium, 1 × 10^5^ HCC cells were inoculated in the upper chamber with or without Matrigel, and 600 µL of 20% FBS medium was added to the lower chamber. Noninvasive cells remaining in the upper chamber were removed after 24 h of incubation. The HCC cells attached to the polycarbonate membrane were fixed with 4% paraformaldehyde, stained with 0.5% crystal violet solution, and photographed using a 10 × microscope. ImageJ was used to count the cells and perform statistical analysis.

### The biological functions and signaling pathways of genes co-expressed with SH2D1A

The genes co-expressed with SH2D1A were obtained using correlation analysis and were defined as genes whose expression was strongly correlated with that of SH2D1A (a correlation coefficient of 0.4 and *P* < 0.001) [2, 8]. The gene ontology annotations include biological processes, molecular functions, and cellular components [9]. The functions and pathways associated with the 567 co-expressed genes of SH2D1A were identified through gene ontology (GO) and KEGG analysis, and the filtering standard was set to adjusted *P* < 0.05.

### Analysis of the immune microenvironment

The expression of the components of the immune microenvironment in HCC tissues was scored through the ssGSEA and ESTIMATE methods. The relationships between the SH2D1A level and the ESTIMATE score, immune score, stromal score, and proportions of immune cells in HCC were determined through relevant analyses. SH2D1A gene expression data were entered into the gene module of the TIMER database to assess the relationships among SH2D1A expression, tumor purity, and the proportions of immune-infiltrating cells.

### Analysis of the expression of SH2D1A and cell markers

In the relevant research module of the TIMER database, the relationship between SH2D1A levels and immune cell markers was explored by relevant methods under non-tumor purity and tumor purity conditions. The data of cell markers in HCC tissues were extracted from the TCGA database, and relevant methods were used to explore the relationship between the SH2D1A level and the levels of cell markers in HCC.

### Statistical analysis

A t test was used to explore the potential significance of differences between the levels of cell markers and the proliferation, migration and invasion rates in SH2D1A expression groups (control group and SH2D1A overexpression groups). Pearson correlation analysis was used to elucidate the relationship of SH2D1A expression with the immune microenvironment components and immune markers. *P* < 0.05 was considered to indicate a significant difference.

## Results

### Elevation of SH2D1A expression promotes cell growth and migration in HCC

**Fig. 1:**
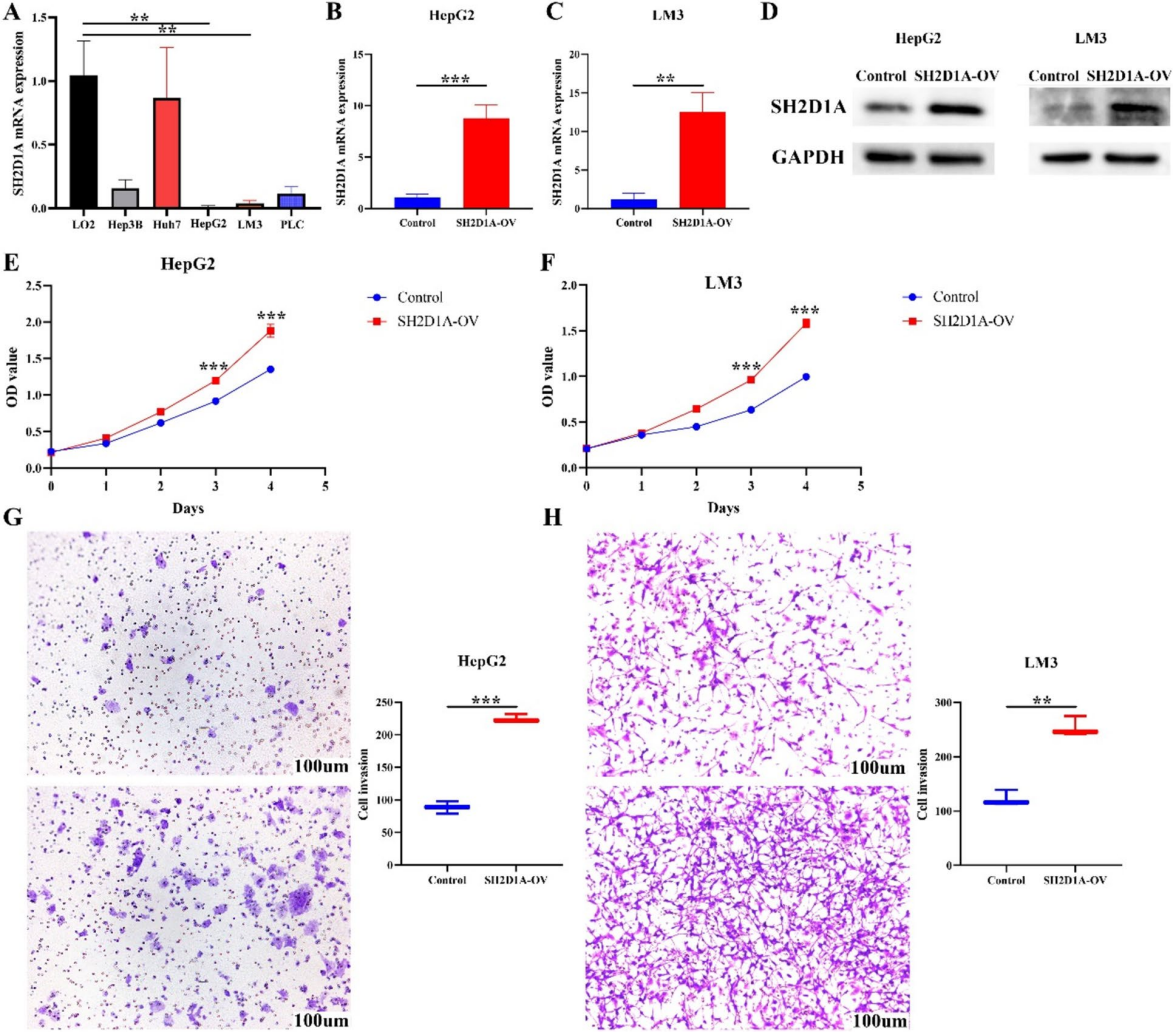
Increased expression of SH2D1A promotes the proliferation and invasion of HCC cells. **A** The expression levels of SH2D1A in normal liver and HCC cells; **B**-**D** Verification of cell models using PCR and western blotting; **E**–**F** cell growth analysis using CCK-8 assay; **G**-**H** cell invasion. Note: HCC, hepatocellular carcinoma; **, *P* < 0.01; ***, *P* < 0.001

**Fig. 2:**
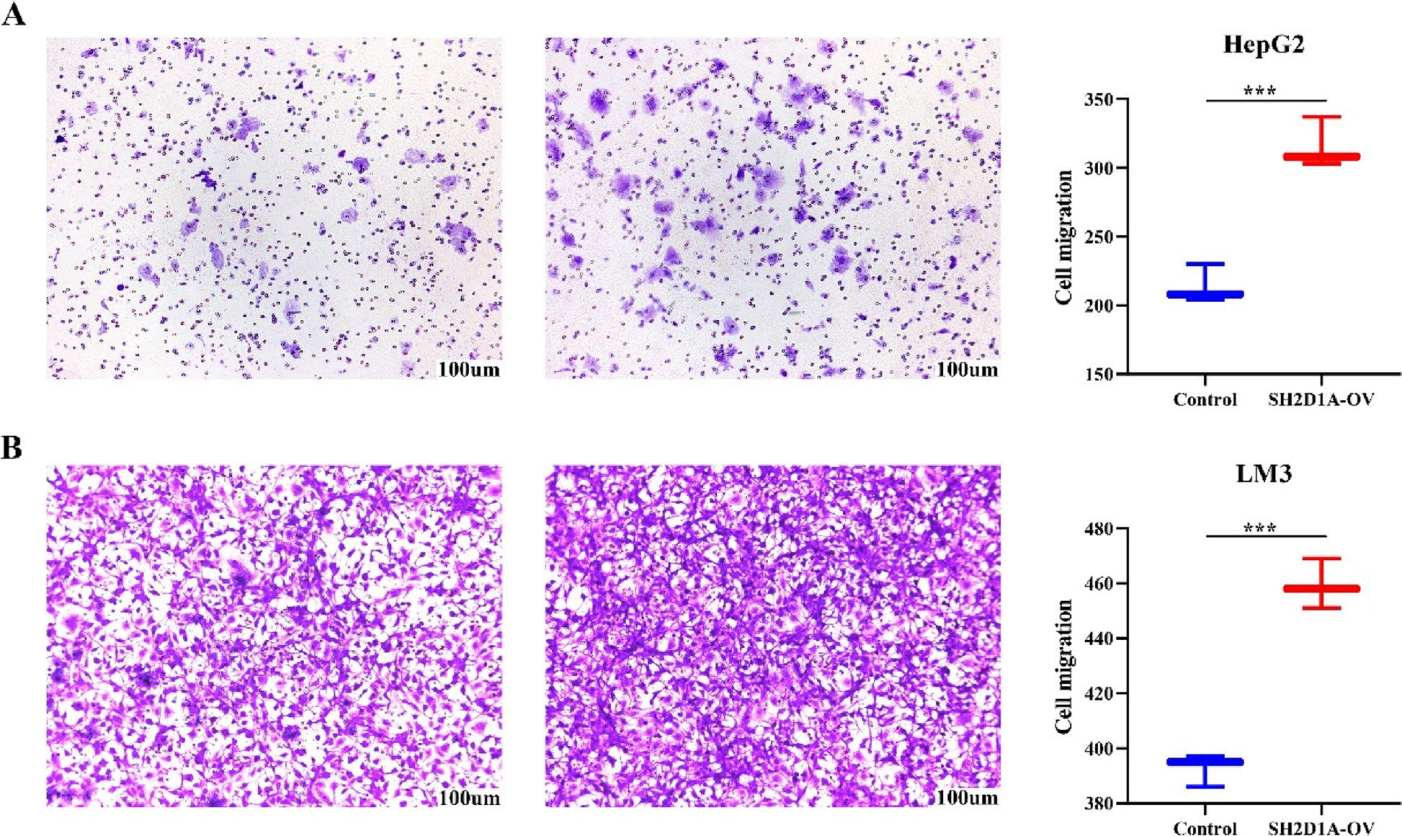
Increased expression of SH2D1A promotes the cell migration of HCC. **A** HepG2 cells; **B** LM3 cells. Note: HCC, hepatocellular carcinoma; ***, *P* < 0.001

Compared with that in normal LO2 hepatocytes, the expression of SH2D1A in HCC cells was significantly decreased (Fig. 1A). We successfully constructed the SH2D1A-overexpressing HCC cell model using PCR and western blotting (Fig. 1B-D). In the cell models, the elevation of SH2D1A expression was significantly positively associated with HCC cell proliferation at 72 and 96 h (Fig. 1E and F). The enhancement of SH2D1A expression significantly promoted HCC cell invasion and migration (Figs. 1G-H and 2).

### Functions and pathways of SH2D1A co-expressed genes

**Fig. 3:**
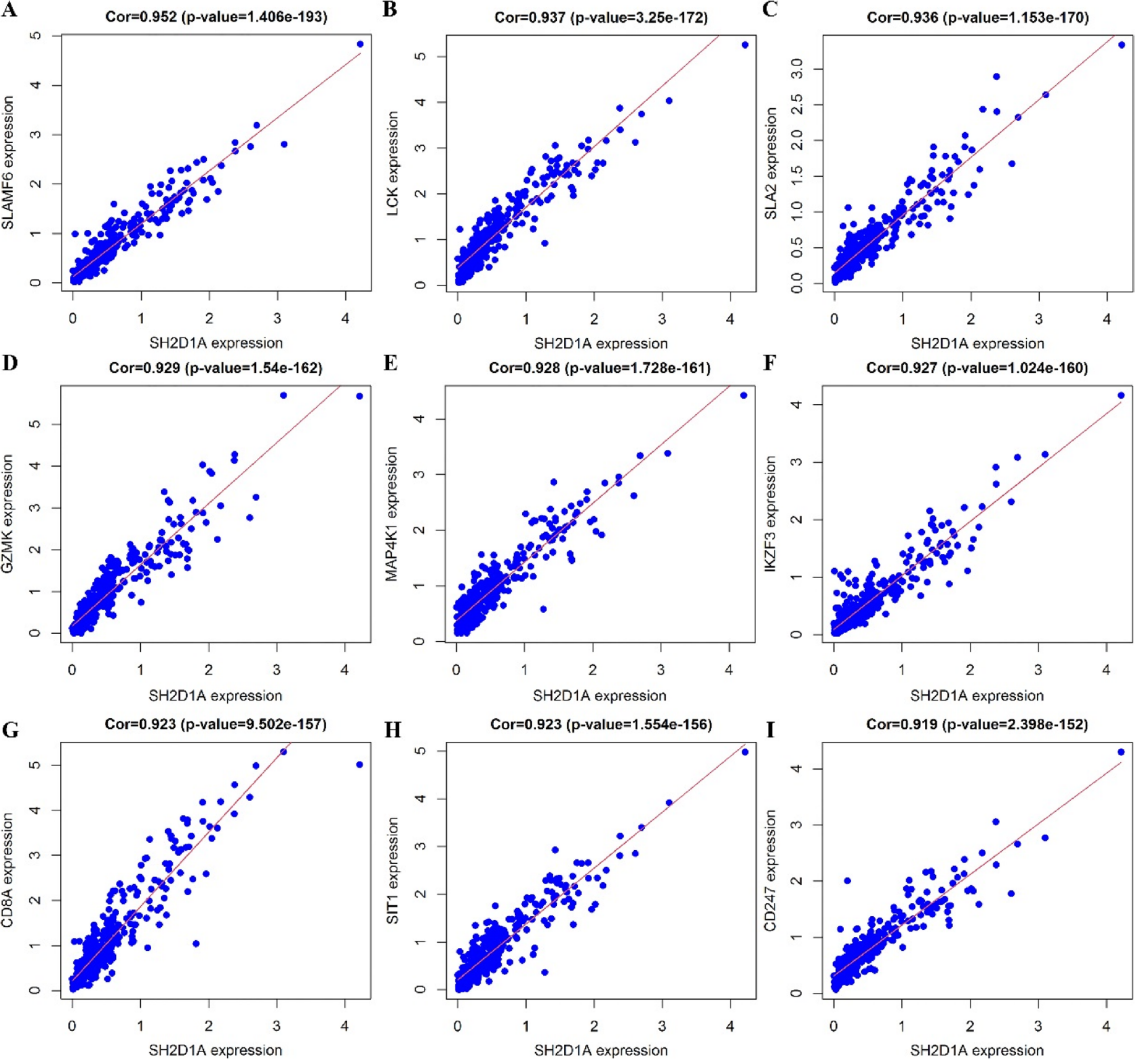
Nine genes co-expressed with SH2D1A in HCC tissues are shown. **A** SLAMF6; **B** LCK; **C** SLA2; **D** GZMK; **E** MAP4K1; **F** IKZF3; **G** CD8A; **H** SIT1; **G** CD247. Note: HCC, hepatocellular carcinoma

**Fig. 4:**
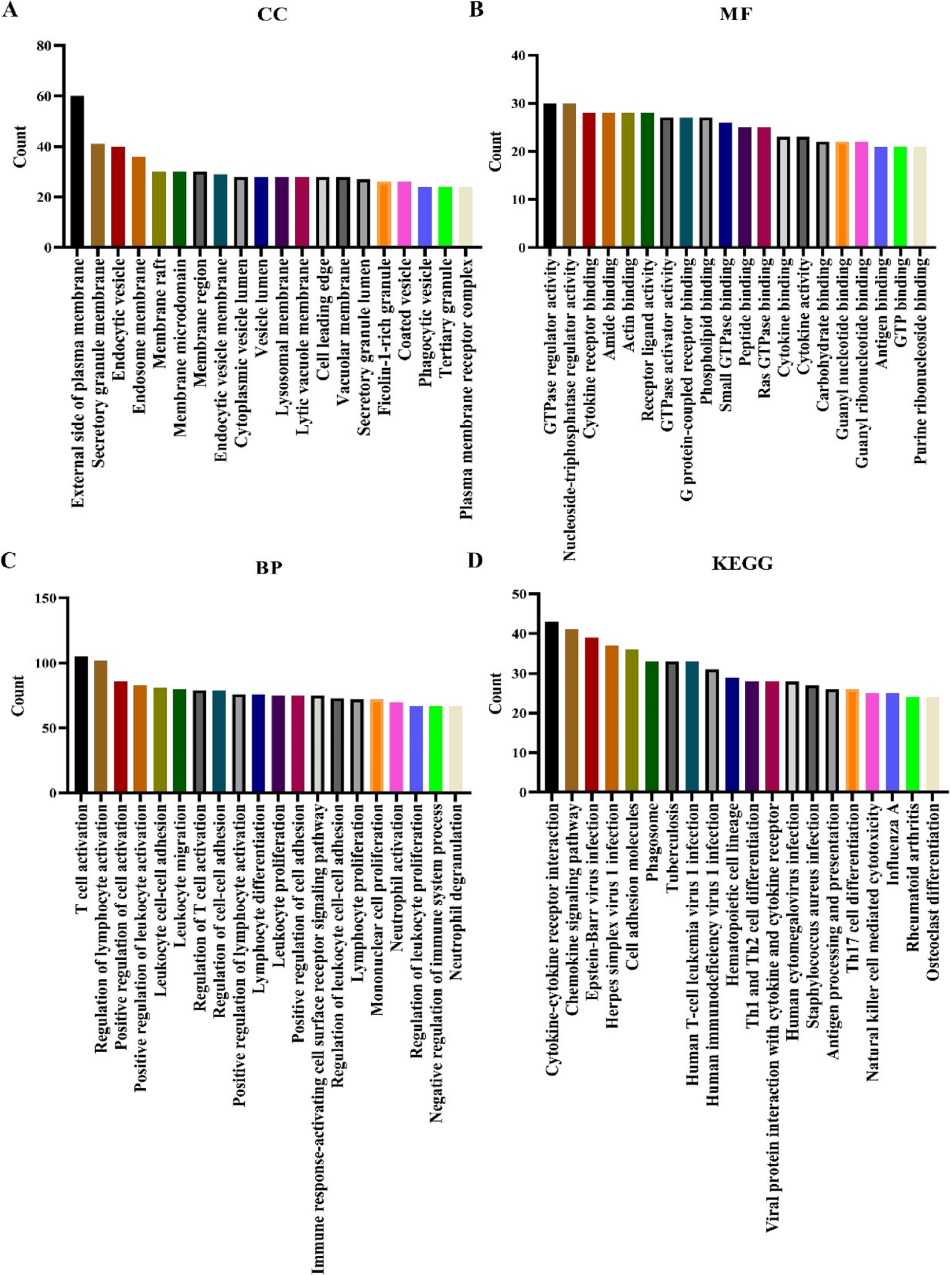
Functions and mechanisms of genes co-expressed with SH2D1A. **A** CC; **B** MF; **C** BP; **D** KEGG. Note: CC, cellular component; BP, biological process; MF, molecular function; KEGG, Kyoto Encyclopedia of Genes and Genomes

**Fig. 5:**
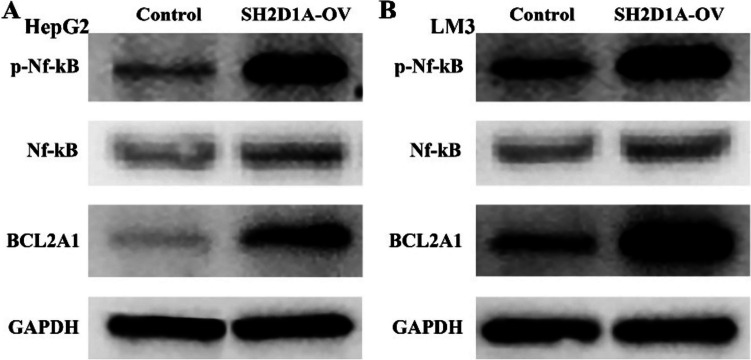
Increased expression of SH2D1A is related to the abnormal pathway of Nf-κB in HCC. Note: HCC, hepatocellular carcinoma

Five hundred and sixty-seven genes co-expressed with SH2D1A were identified based on data obtained from experiments performed using HCC tissues (Fig. 3 and Table S1). The genes co-expressed with SH2D1A were associated with leukocyte cell‒cell adhesion, cell proliferation, migration, T-cell differentiation, granulocyte migration, B-cell activation, cell death, and others (Fig. 4A-C and Table S2) and were involved in pathways involving chemokines, B-cell receptors, NF-κB, T-cell receptors, PD-L1, PD-1, and other factors (Fig. 4D and Table 1). In addition, we found via western blotting that SH2D1A overexpression significantly increased p-Nf-κB and BCL2A1 protein levels but had no the significant effect on Nf-κB protein in HCC cells (Fig. 5).

**Table 1:** Pathways of SH2D1A co-expressed genes

ID	Description	P	Adjust P
hsa04062	Chemokine signaling pathway	7.00E-20	1.69E-17
hsa04514	Cell adhesion molecules	1.93E-19	2.32E-17
hsa04640	Hematopoietic cell lineage	2.45E-18	1.87E-16
hsa04658	Th1 and Th2 cell differentiation	3.10E-18	1.87E-16
hsa04612	Antigen processing and presentation	4.00E-18	1.93E-16
hsa05330	Allograft rejection	1.60E-17	6.41E-16
hsa05169	Epstein-Barr virus infection	2.48E-17	8.53E-16
hsa04061	Viral protein interaction with cytokine and cytokine receptor	3.64E-17	1.10E-15
hsa05150	Staphylococcus aureus infection	1.21E-16	3.23E-15
hsa05416	Viral myocarditis	1.58E-16	3.80E-15
hsa05332	Graft-versus-host disease	1.75E-16	3.84E-15
hsa04145	Phagosome	2.15E-16	4.31E-15
hsa04940	Type I diabetes mellitus	3.03E-16	5.61E-15
hsa05320	Autoimmune thyroid disease	2.03E-15	3.50E-14
hsa05140	Leishmaniasis	5.59E-15	8.98E-14
hsa04659	Th17 cell differentiation	2.18E-14	3.20E-13
hsa04060	Cytokine-cytokine receptor interaction	2.26E-14	3.20E-13
hsa05152	Tuberculosis	4.01E-14	5.36E-13
hsa05323	Rheumatoid arthritis	5.33E-14	6.76E-13
hsa05340	Primary immunodeficiency	1.82E-13	2.20E-12
hsa05321	Inflammatory bowel disease	2.14E-12	2.46E-11
hsa05166	Human T-cell leukemia virus 1 infection	1.20E-11	1.31E-10
hsa04650	Natural killer cell mediated cytotoxicity	2.28E-11	2.39E-10
hsa05310	Asthma	3.83E-11	3.84E-10
hsa04380	Osteoclast differentiation	8.60E-11	8.29E-10
hsa05170	Human immunodeficiency virus 1 infection	1.15E-10	1.06E-09
hsa04672	Intestinal immune network for IgA production	2.29E-10	2.04E-09
hsa05145	Toxoplasmosis	1.35E-09	1.16E-08
hsa04662	B cell receptor signaling pathway	1.47E-09	1.22E-08
hsa04064	NF-kappa B signaling pathway	2.12E-09	1.70E-08
hsa05164	Influenza A	7.97E-09	6.20E-08
hsa05135	Yersinia infection	1.12E-08	8.46E-08
hsa04660	T cell receptor signaling pathway	1.32E-08	9.63E-08
hsa05163	Human cytomegalovirus infection	3.73E-08	2.65E-07
hsa05235	PD-L1 expression and PD-1 checkpoint pathway in cancer	3.92E-08	2.70E-07
hsa05322	Systemic lupus erythematosus	1.07E-06	7.19E-06
hsa04621	NOD-like receptor signaling pathway	1.71E-06	1.11E-05
hsa05167	Kaposi sarcoma-associated herpesvirus infection	5.00E-06	3.17E-05
hsa04625	C-type lectin receptor signaling pathway	1.00E-05	6.19E-05
hsa05142	Chagas disease	3.51E-05	0.000211572
hsa04630	JAK-STAT signaling pathway	5.18E-05	0.000302854
hsa04664	Fc epsilon RI signaling pathway	5.28E-05	0.000302854
hsa04666	Fc gamma R-mediated phagocytosis	8.59E-05	0.000481381
hsa05162	Measles	8.85E-05	0.000484625
hsa05168	Herpes simplex virus 1 infection	9.06E-05	0.000485131
hsa04620	Toll-like receptor signaling pathway	0.000176672	0.000925606
hsa04610	Complement and coagulation cascades	0.000408532	0.002094811
hsa05202	Transcriptional misregulation in cancer	0.000449467	0.002256701
hsa05144	Malaria	0.000602872	0.002965145
hsa04611	Platelet activation	0.000989296	0.004768407
hsa04670	Leukocyte transendothelial migration	0.001482486	0.007005474
hsa05133	Pertussis	0.002547957	0.011808801
hsa04623	Cytosolic DNA-sensing pathway	0.00281258	0.01278928
hsa04668	TNF signaling pathway	0.003979552	0.017410689
hsa05161	Hepatitis B	0.004034692	0.017410689
hsa05130	Pathogenic Escherichia coli infection	0.004045637	0.017410689
hsa05221	Acute myeloid leukemia	0.004144591	0.017523621
hsa04217	Necroptosis	0.008674073	0.036042268
hsa04072	Phospholipase D signaling pathway	0.012189773	0.049792125

### SH2D1A is associated with the HCC immune microenvironment

**Fig. 6:**
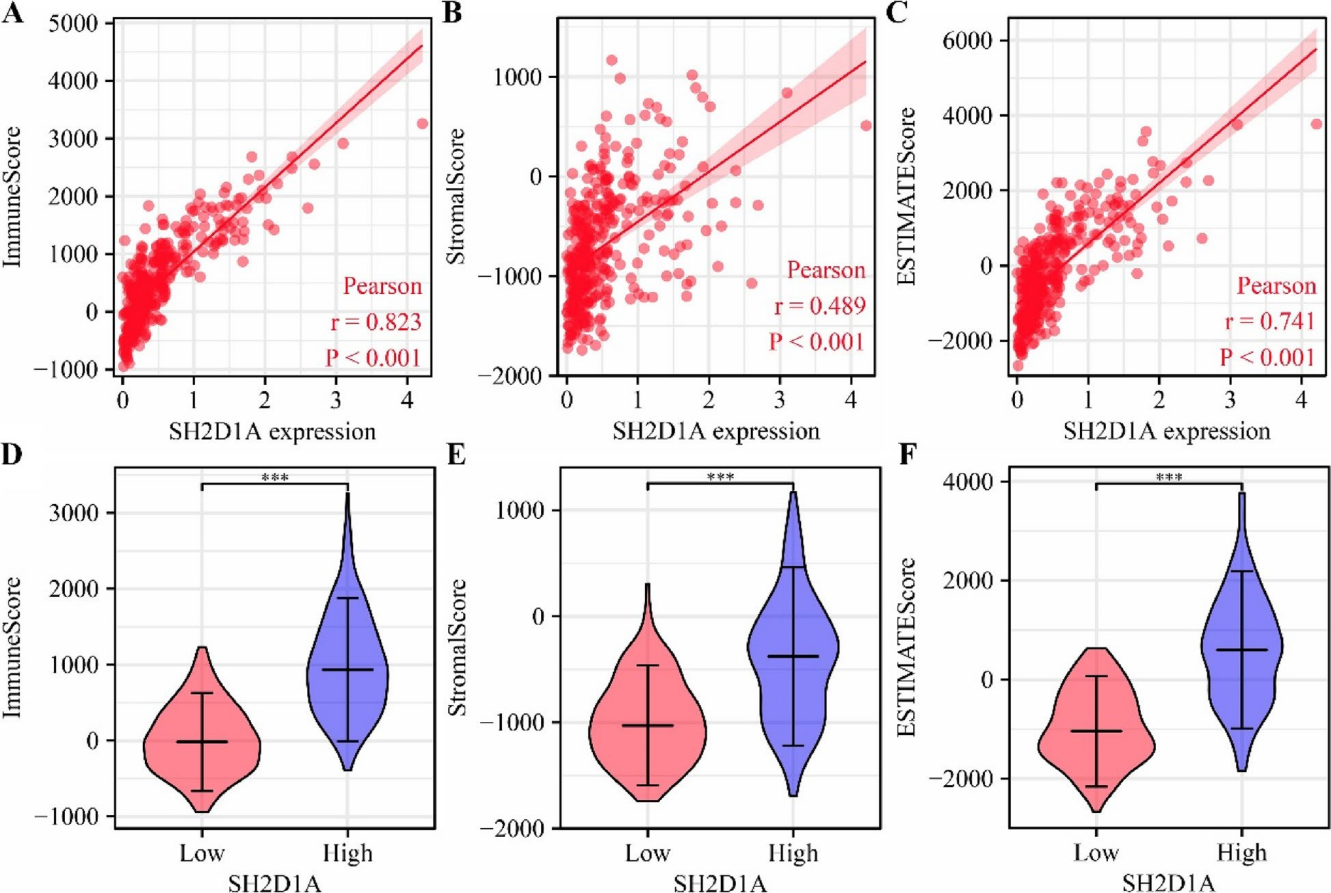
SH2D1A expression is significantly correlated with the immune, stromal, and ESTIMATE scores in TCGA database. **A**-**C** SH2D1A expression is correlated with the immune score, stromal score, and ESTIMATE score. **D**-**F** The immune score, stromal score, and ESTIMATE score are different in the high- and low-SH2D1A expression groups

**Fig. 7:**
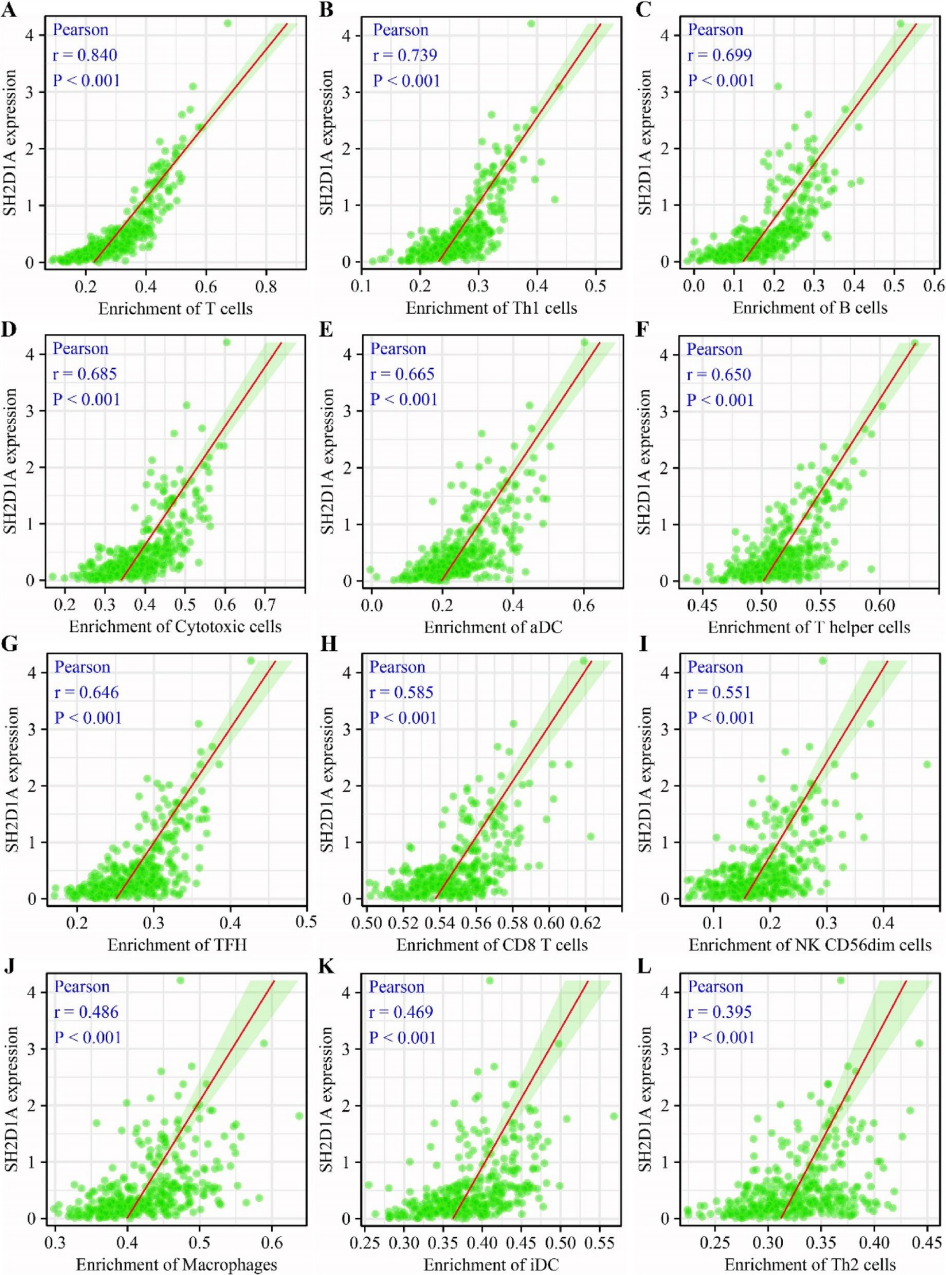
SH2D1A expression is significantly correlated with immune cell abundance, according to data from the TCGA database. **A** T cells; **B** Th1 cells; **C** B cells; **D** cytotoxic cells; **E** aDCs; **F** T helper cells; **G** TFHs; **H** CD8^+^ T cells; **I** NK CD56 dim cells; **J** macrophages; **K** iDCs; **L** Th2 cells

Correlation analysis revealed that SH2D1A expression was significantly correlated with the immune score (*r* = 0.823), stromal score (*r* = 0.489), and ESTIMATE score (*r* = 0.741) (Fig. 6A-C). In the high- and low-SH2D1A expression groups, the immune score, stromal score, and ESTIMATE score were significantly different (Fig. 6D-F). The SH2D1A expression level was significantly correlated with aDC (*r* = 0.665), B-cell (*r* = 0.699), CD8^+^ T-cell (*r* = 0.585), cytotoxic cell (*r* = 0.685), DC (*r* = 0.312), eosinophil (*r* = 0.202), iDC (*r* = 0.469), macrophage (*r* = 0.486), mast cell (*r* = 0.194), neutrophil (*r* = 0.278), NK CD56 bright cell (*r* = 0.205), NK CD56 dim cell (*r* = 0.551), NK cell (*r* = 0.203), pDC (*r* = 0.148), T-cell (*r* = 0.840), T helper cell (*r* = 0.650), Tcm (*r* = 0.167), Tem (*r* = 0.373), TFH (*r* = 0.646), Tgd (*r* = 0.285), Th1 cell (*r* = 0.739), Th2 cell (*r* = 0.395), and Treg (*r* = 0.292) levels (Fig. 7 and S1).

**Fig. 8:**
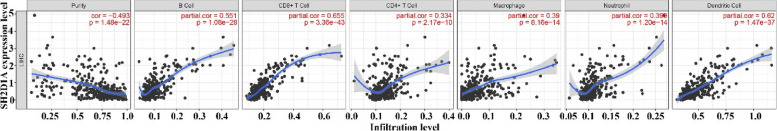
SH2D1A expression is significantly correlated with immune cell abundance, according to data from the TIMER database

**Fig. 9:**
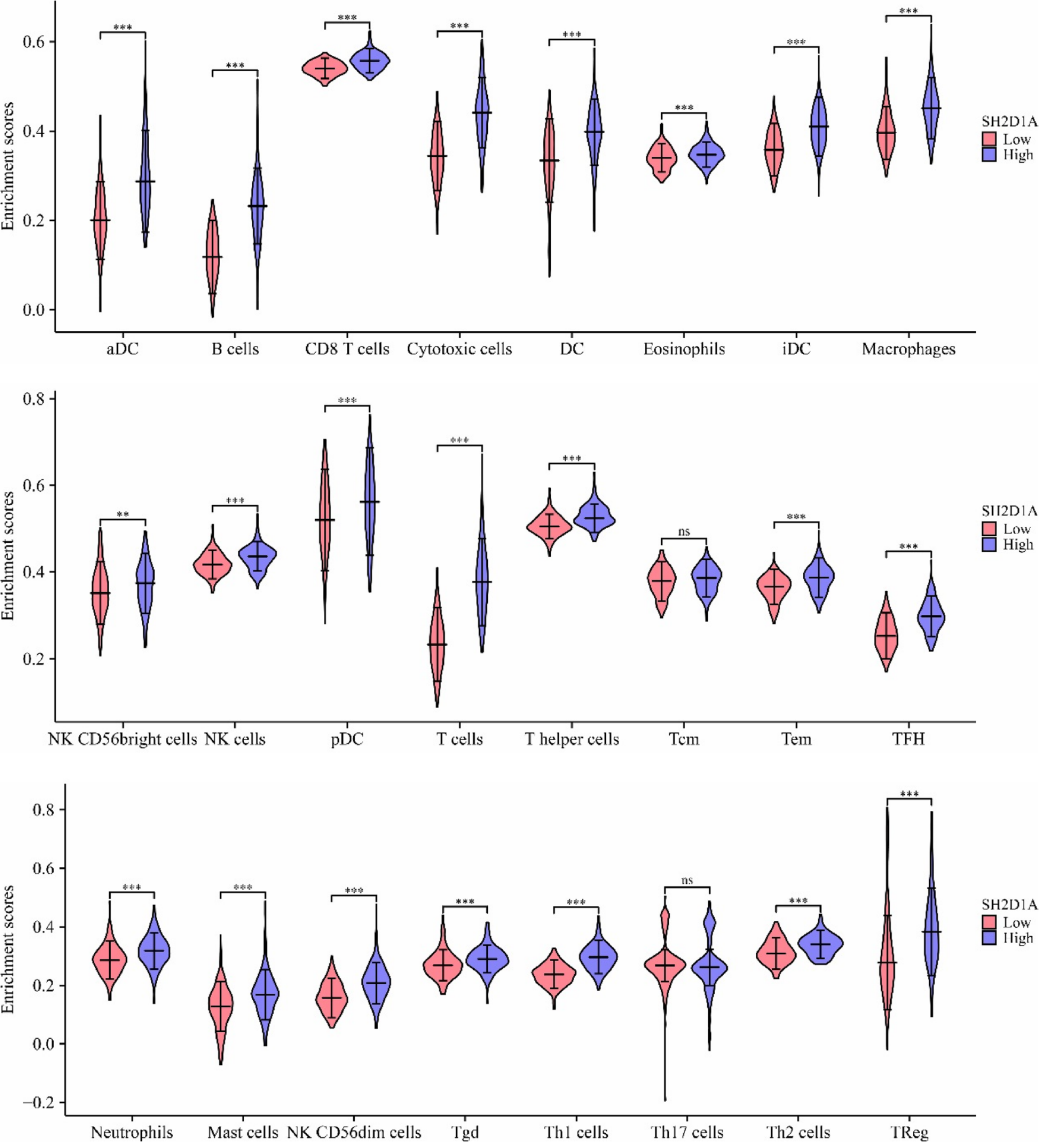
Immune cells in the high- and low-SH2D1A expression groups. Note: **, *P* < 0.01; ***, *P* < 0.001; ns, no statistical significance

In the TIMER database, SH2D1A expression was found to be significantly correlated with HCC purity and the levels of B cells, CD8^+^ T cells, CD4^+^ T cells, macrophages, neutrophils, and dendritic cells (Fig. 8). In the high- and low- SH2D1A expression groups, the levels of aDCs, B cells, CD8^+^ T cells, cytotoxic cells, DCs, eosinophils, iDCs, macrophages, NK CD56 bright cells, NK cells, pDCs, T cells, T helper cells, Tems, TFHs, neutrophils, mast cells, NK CD56 dim cells, Tgds, Th1 cells, Th2 cells, and Treg were significantly different (Fig. 9).

### SH2D1A expression correlates with the expression of immune cell markers based on tumor purity

In the TIMER database, in the absence of tumor purity, the expression level of SH2D1A was significantly correlated with the expression of the immune cell markers PDCD1, MS4A4A, HLA-DRA, KIR2DL4, CD8A, CD3E, CD79A, CD86, STAT6, CSF1R, CCL2, CD68, IL10, IRF5, PTGS2, IL17A, CD163, VSIG4, CD8B, CCR7, HLA-DQB1, KIR2DL1, KIR2DL3, KIR3DL2, KIR3DL1, HLA-DPA1, CD19, CD1C, NRP1, CD3D, ITGAX, TBX21, HLA-DPB1, STAT4, STAT1, IFNG, TNF, KIR3DL3, GATA3, STAT5A, IL13, CD2, IL21, STAT3, FOXP3, ITGAM, CCR8, TGFB1, CTLA4, LAG3, KIR2DS4, HAVCR2, and GZMB (Table 2). In addition, SH2D1A expression in tumor purity was found to be significantly correlated with the expression of the immune cell markers VSIG4, CD3E, CD2, TBX21, KIR2DS4, CD3D, CD79A, PDCD1, STAT5B, CTLA4, CCR7, HLA-DPA1, CD163, IFNG, CD86, KIR3DL1, HLA-DRA, LAG3, ITGAX, GZMB, CCR8, STAT4, IL13, HAVCR2, CD19, CD1C, CD8A, TNF, MS4A4A, STAT1, KIR2DL4, IL10, HLA-DQB1, FOXP3, STAT5A, CD8B, KIR3DL2, CD68, CCL2, TGFB1, KIR2DL3, ITGAM, HLA-DPB1, PTGS2, IL21, NRP1, CSF1R, IL17A, STAT6, GATA3, KIR3DL3, STAT3, IRF5, and BCL6 (Table 3).

**Table 2:** The expression level of SH2D1A was significantly correlated with the expression of the immune cell markers in HCC in the absence of tumor purity

Gene	Coefficient	*P*	Gene	Coefficient	*P*
CD8A	0.887016405	0	HLA-DQB1	0.672194662	0
CD8B	0.79381506	1.05E-81	HLA-DRA	0.7109175	0
CD3D	0.784088575	0	HLA-DPA1	0.73305306	0
CD3E	0.919849508	0	CD1C	0.592266849	1.70E-36
CD2	0.896876887	0	NRP1	0.232362638	6.49E-06
CD19	0.620480458	7.44E-41	ITGAX	0.60879195	0
CD79A	0.77372608	3.79E-75	TBX21	0.833910496	2.77E-97
CD86	0.72178471	0	STAT4	0.593114347	0
CSF1R	0.64226918	0	STAT1	0.567067494	0
CCL2	0.502753697	0	IFNG	0.680123851	1.07E-51
CD68	0.492583724	0	TNF	0.615371131	4.94E-40
IL10	0.593359255	1.17E-36	GATA3	0.75729304	2.88E-70
NOS2	0.053431612	0.304690837	STAT6	0.127106478	0.014288533
IRF5	0.177298342	0.000602022	STAT5A	0.474038948	3.50E-22
PTGS2	0.457054407	1.51E-20	IL13	0.168795537	0.00109964
CD163	0.61504048	5.57E-40	BCL6	0.089150885	0.086382304
VSIG4	0.524494932	0	IL21	0.26668931	1.85E-07
MS4A4A	0.636753326	0	STAT3	0.24223252	2.53E-06
CEACAM8	0.086160103	0.09751073	IL17A	0.16280217	0.001653927
ITGAM	0.394698933	1.72E-15	FOXP3	0.466619616	1.86E-21
CCR7	0.749726507	3.78E-68	CCR8	0.628091839	4.15E-42
KIR2DL1	0.139414926	0.007157941	STAT5B	0.093033743	0.07349801
KIR2DL3	0.338216741	2.22E-11	TGFB1	0.441941359	0
KIR2DL4	0.518825565	5.93E-27	PDCD1	0.746149813	3.57E-67
KIR3DL1	0.295178969	6.79E-09	CTLA4	0.748133559	1.03E-67
KIR3DL2	0.442756938	3.05E-19	LAG3	0.626631057	0
KIR3DL3	0.174882461	0.000716424	HAVCR2	0.67277581	0
KIR2DS4	0.28554748	2.16E-08	GZMB	0.636271347	0
HLA-DPB1	0.726406634	0			

Note: *HCC *Hepatocellular carcinoma

**Table 3:** The expression level of SH2D1A was significantly correlated with the expression of immune cell markers in HCC with tumor purity

Gene	Coefficient	P	Gene	Coefficient	P
CD8A	0.85456309	1.27E-99	HLA-DQB1	0.583105318	8.25E-33
CD8B	0.743393609	7.09E-62	HLA-DRA	0.621319185	3.20E-38
CD3D	0.731080308	6.62E-59	HLA-DPA1	0.655690032	9.07E-44
CD3E	0.894548317	5.35E-122	CD1C	0.519231383	3.28E-25
CD2	0.86471045	1.31E-104	NRP1	0.184854906	0.000558818
CD19	0.547172959	2.46E-28	ITGAX	0.493683486	1.35E-22
CD79A	0.701937989	1.73E-52	TBX21	0.794043823	3.79E-76
CD86	0.62316122	1.68E-38	STAT4	0.558224737	1.18E-29
CSF1R	0.524678418	8.50E-26	STAT1	0.543002049	7.52E-28
CCL2	0.340264645	8.47E-11	IFNG	0.642013885	1.78E-41
CD68	0.354422898	1.20E-11	TNF	0.512228684	1.80E-24
IL10	0.462535122	1.08E-19	GATA3	0.680882767	2.55E-48
NOS2	0.012074031	0.823177975	STAT6	0.152140349	0.004623899
IRF5	0.197750673	0.000218772	STAT5A	0.417545542	5.48E-16
PTGS2	0.286609443	6.02E-08	IL13	0.179179251	0.00082841
CD163	0.498529481	4.48E-23	BCL6	0.123503491	0.021765188
VSIG4	0.374536414	6.24E-13	IL21	0.243626764	4.70E-06
MS4A4A	0.509326765	3.59E-24	STAT3	0.14196436	0.008273776
CEACAM8	0.051908934	0.336393954	IL17A	0.167942121	0.001746115
ITGAM	0.295974154	2.10E-08	FOXP3	0.443137012	5.01E-18
CCR7	0.677354884	1.18E-47	CCR8	0.565261309	1.61E-30
KIR2DL1	0.099904974	0.063802467	STAT5B	0.224855069	2.49E-05
KIR2DL3	0.306235488	6.34E-09	TGFB1	0.310151688	3.96E-09
KIR2DL4	0.487657097	5.20E-22	PDCD1	0.694787235	4.94E-51
KIR3DL1	0.269862725	3.60E-07	CTLA4	0.690286203	3.88E-50
KIR3DL2	0.388988451	6.57E-14	LAG3	0.60025003	3.79E-35
KIR3DL3	0.142759694	0.007915913	HAVCR2	0.556233637	2.06E-29
KIR2DS4	0.327847865	4.36E-10	GZMB	0.570231739	3.83E-31
HLA-DPB1	0.64683208	2.85E-42			

*HCC* Hepatocellular carcinoma

### Verification of the relationship between SH2D1A and immune cell markers

**Fig. 10:**
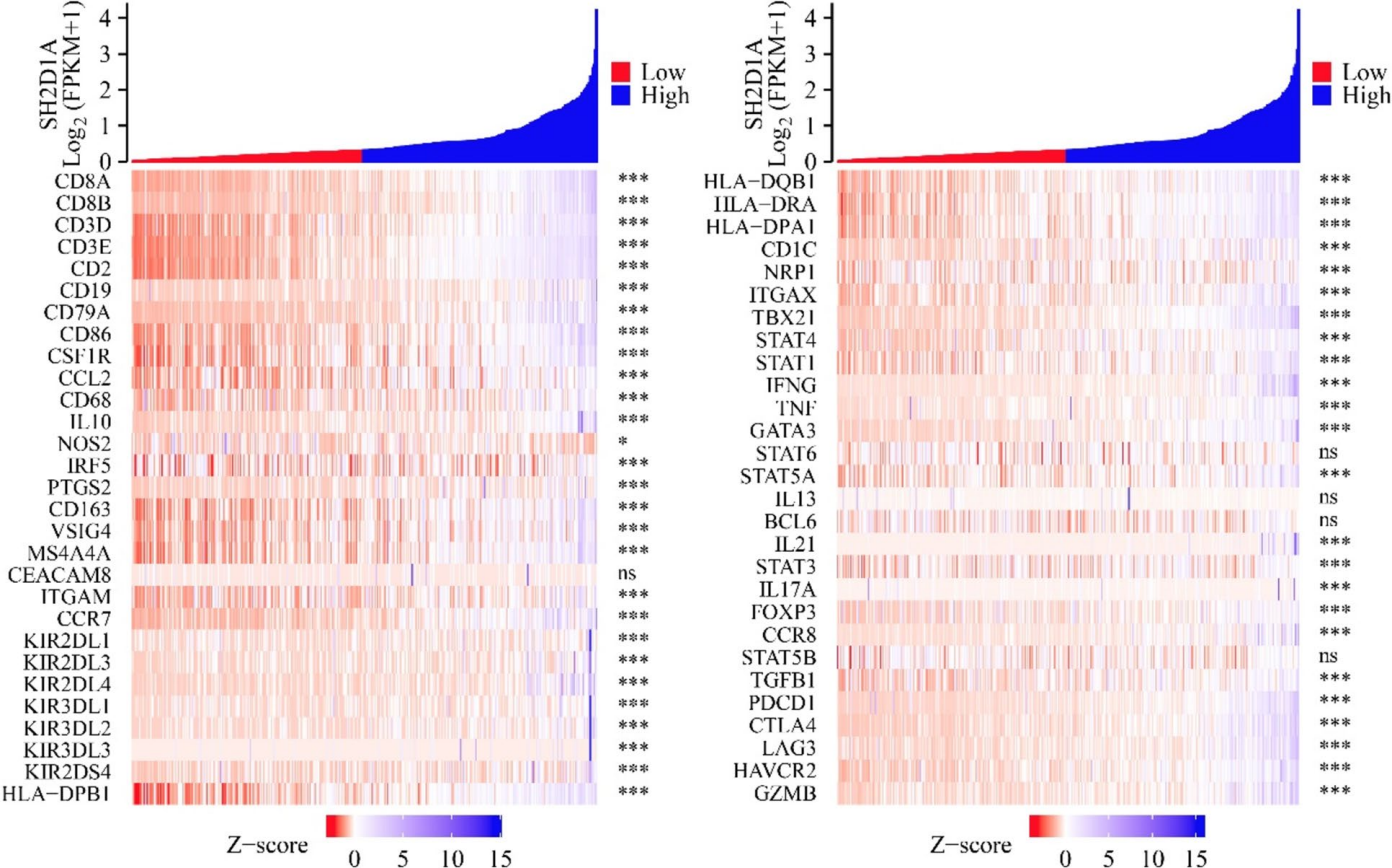
SH2D1A expression was significantly correlated with the expression of immune cell markers in HCC. Note: HCC, hepatocellular carcinoma; *, *P* < 0.05; ***, *P* < 0.001; ns, no statistical significance

In the TCGA database, the expression level of SH2D1A was significantly correlated with the expression of the immune cell markers ITGAM, HLA-DPB1, CD3D, KIR3DL3, PTGS2, KIR3DL2, VSIG4, KIR3DL1, KIR2DL4, KIR3DL1, KIR2DL3, CCR7, KIR2DL1, CEACAM8, CD163, IRF5, CD79A, NOS2, IL10, CD68, MS4A4A, CCL2, CSF1R, CD86, CD19, KIR2DS4, CD2, CD3E, CD8B, CD8A, and PDCD1 (Fig. 10).

## Discussion

Evidence from studies has shown that the inhibition of oncogene expression can delay the progression of HCC, which can improve the prognosis of patients with HCC [10–12]. For example, ATG14 expression is significantly elevated in HCC tissues and cells and indicates a poor prognosis in patients with HCC. Inhibition of ATG14 expression attenuates HCC cell proliferation and migration. XIST can promote autophagy by promoting the expression of ATG14 [10]. In addition, SH2D1A mutations are associated with disease progression [13–16], while the roles of SH2D1A in HCC and other cancers have not been identified. We found that genes co-expressed with SH2D1A are associated with leukocyte cell‒cell adhesion, cell proliferation, migration, T-cell differentiation, granulocyte migration, B-cell activation, and cell death. In addition, increased expression of SH2D1A can promote cell proliferation, cell invasion and migration in HCC cell models according to CCK-8 and Transwell assays.

Recent studies have indicated that abnormal gene expression and Nf-κB signaling mechanisms may be involved in the progression of cancer [17–21]. For example, the expression levels of CD13 are higher in metastatic HCC tissues than in primary HCC tissues. Overexpression of CD13 predicts a poor prognosis in HCC patients. CD13 can promote the proliferation, invasion, cell cycle progression and sorafenib resistance of HCC cells, which is consistent with the finding that HDAC5-LSD1-Nf-κB is related to the activation of carcinogenic signaling [19]. The increased expression of ZNF545 can inhibit the proliferation, migration and invasion of HCC cells, induce the G1/S phase arrest and apoptosis of SNU449 and Huh7 cells, and inhibit the tumor growth of SNU449 cell-xenografted mice, which is related to the inactivation of the Nf-κB signaling pathway [21]. In our study, SH2D1A was associated with the Nf-κB signaling pathway according to KEGG analysis, and SH2D1A overexpression significantly increased p-Nf-κB and BCL2A1 protein levels in HCC cell models. In addition, BCL2A1 is a member of the Nf-κB signaling pathway. Therefore, preliminary evidence suggests that SH2D1A may promote HCC progression through the Nf-κB signaling pathway.

The immune microenvironment contributes to HCC progression [22–25]. PD-1 inhibitor therapy is well tolerated and has potential clinical benefits as a first-line therapy. The median OS of patients with HCC was 6.6 months, the PFS was 5.3 months, and the overall response rate (ORR) was 30.8%. Moreover, one patient achieved a complete response [24]. SH2D1A expression was found to be associated with the HCC immune microenvironment in our study. SH2D1A expression was found to be correlated with the immune score, stromal score, ESTIMATE score, and levels of B cells, CD8^+^ T cells, T cells, T helper cells, Tfh cells, Th1 cells, Th2 cells, Tregs, and other cells and was associated with the progression of the disease. The expression levels of SH2D1A were significantly correlated with the levels of the immune cell markers CD3E, CD2, CD8A, CD8B, CD3D, CD79A, PDCD1, CTLA4, and others.

Based on our research, we first reported the role of SH2D1A in the progression of HCC and its potential signaling mechanism, providing a new theoretical basis for the treatment of HCC patients. However, this study still has some limitations. In the future, we need to explore the expression levels of SH2D1A in HCC tissues and the relationship between SH2D1A overexpression and poor prognosis and clinicopathological features of HCC patients. In addition, we should identify the targets of SH2D1A through bioinformatics analysis and RNA sequencing to further understand the signaling mechanisms of SH2D1A in HCC cell models.

## Conclusion

SH2D1A overexpression promotes cancer cell growth and metastasis via the Nf-κB signaling pathway and is significantly related to the immune microenvironment in HCC. These results show that SH2D1A is a therapeutic target for HCC patients.

### Supplementary Information

**Additional file 1: Figure S1.** SH2D1A expression is significantly correlated with immune cell abundance according to data from the TCGA database. **Table S1.** Five hundred and sixty-seven SH2D1A co-expressed genes. **Table S2.** The functions of SH2D1A co-expressed genes.

**Additional file 2. **

## Data Availability

The data are available in the TCGA (https://portal.gdc.cancer.gov/) database and can be downloaded directly.
