# Supplementary material for: Overexpression of SH2D1A promotes cancer progression and is associated with immune cell infiltration in hepatocellular carcinoma via bioinformatics and in vitro study
Source: BMC Cancer. 2023 Oct 19;23:1005. doi: 10.1186/s12885-023-11315-1 (PMC10585762; doi:10.1186/s12885-023-11315-1)
Supplement: Supplementary file 1 — Additional file 1: Figure S1. SH2D1A expression is significantly correlated with immune cell abundance according to data from the TCGA database. Table S1. Five hundred and sixty-seven SH2D1A co-expressed genes. Table S2. The functions of SH2D1A co-expressed genes. [file 12885_2023_11315_MOESM1_ESM.docx]

**Supplementary materials**


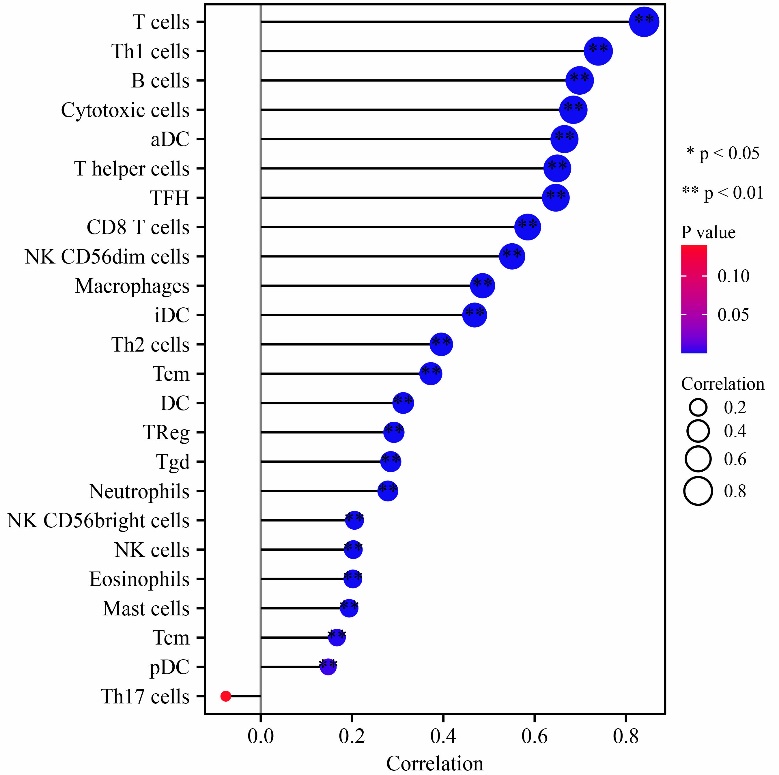


Figure S1. SH2D1A expression is significantly correlated with immune cell abundance according to data from the TCGA database.

**Table S.**

Table S1. Five hundred and sixty-seven SH2D1A co-expressed genes.

| Gene1 | Gene2 | Coefficient | P value |
| --- | --- | --- | --- |
| SH2D1A | LAIR1 | 0.614 | 3.84E-40 |
| SH2D1A | S100B | 0.524 | 9.60E-28 |
| SH2D1A | CRLF3 | 0.428 | 4.14E-18 |
| SH2D1A | CSF2RB | 0.72 | 5.62E-61 |
| SH2D1A | JAK2 | 0.551 | 4.23E-31 |
| SH2D1A | GBP5 | 0.868 | 4.53E-115 |
| SH2D1A | SAMD9 | 0.535 | 4.25E-29 |
| SH2D1A | PSTPIP1 | 0.851 | 2.56E-106 |
| SH2D1A | LST1 | 0.673 | 1.05E-50 |
| SH2D1A | PLA2G7 | 0.472 | 3.91E-22 |
| SH2D1A | SAMD9L | 0.657 | 1.40E-47 |
| SH2D1A | CCDC88C | 0.465 | 1.71E-21 |
| SH2D1A | FMNL3 | 0.5 | 5.20E-25 |
| SH2D1A | SIGLEC10 | 0.684 | 6.21E-53 |
| SH2D1A | MTHFD2 | 0.525 | 7.65E-28 |
| SH2D1A | ADAMDEC1 | 0.701 | 1.29E-56 |
| SH2D1A | EMP3 | 0.54 | 1.15E-29 |
| SH2D1A | HLA-B | 0.423 | 1.23E-17 |
| SH2D1A | CETP | 0.42 | 2.21E-17 |
| SH2D1A | BIN2 | 0.878 | 2.53E-121 |
| SH2D1A | TAP2 | 0.529 | 2.17E-28 |
| SH2D1A | CPNE5 | 0.596 | 2.53E-37 |
| SH2D1A | LAMP3 | 0.458 | 7.90E-21 |
| SH2D1A | GNA15 | 0.596 | 2.48E-37 |
| SH2D1A | TRIM69 | 0.495 | 1.70E-24 |
| SH2D1A | GNG2 | 0.734 | 1.38E-64 |
| SH2D1A | APOL4 | 0.48 | 5.89E-23 |
| SH2D1A | HCLS1 | 0.779 | 2.56E-77 |
| SH2D1A | ABR | 0.401 | 7.39E-16 |
| SH2D1A | CD38 | 0.712 | 5.10E-59 |
| SH2D1A | LRRC8C | 0.551 | 4.55E-31 |
| SH2D1A | ADAP2 | 0.598 | 1.22E-37 |
| SH2D1A | NAGK | 0.544 | 3.54E-30 |
| SH2D1A | S1PR4 | 0.839 | 1.73E-100 |
| SH2D1A | TNFSF13B | 0.732 | 4.38E-64 |
| SH2D1A | MAX | 0.462 | 3.71E-21 |
| SH2D1A | IFI16 | 0.548 | 1.14E-30 |
| SH2D1A | GBP1 | 0.546 | 1.78E-30 |
| SH2D1A | PIP4K2A | 0.525 | 6.55E-28 |
| SH2D1A | RGS10 | 0.605 | 9.41E-39 |
| SH2D1A | GSTP1 | 0.447 | 8.28E-20 |
| SH2D1A | PTPN7 | 0.903 | 1.13E-138 |
| SH2D1A | GIMAP2 | 0.512 | 2.38E-26 |
| SH2D1A | RALGDS | 0.427 | 4.82E-18 |
| SH2D1A | GLIPR2 | 0.605 | 1.21E-38 |
| SH2D1A | ARPC2 | 0.441 | 2.98E-19 |
| SH2D1A | PILRA | 0.683 | 8.70E-53 |
| SH2D1A | ARHGAP25 | 0.867 | 9.14E-115 |
| SH2D1A | GIMAP8 | 0.545 | 2.80E-30 |
| SH2D1A | LBH | 0.51 | 3.98E-26 |
| SH2D1A | EMILIN2 | 0.491 | 4.38E-24 |
| SH2D1A | ITGB2 | 0.736 | 4.94E-65 |
| SH2D1A | TLR2 | 0.412 | 8.68E-17 |
| SH2D1A | HK3 | 0.659 | 5.18E-48 |
| SH2D1A | IRF9 | 0.422 | 1.53E-17 |
| SH2D1A | CD37 | 0.802 | 2.92E-85 |
| SH2D1A | PDCD1LG2 | 0.73 | 1.51E-63 |
| SH2D1A | SELL | 0.738 | 1.19E-65 |
| SH2D1A | FYN | 0.52 | 2.53E-27 |
| SH2D1A | SERPINB9 | 0.497 | 1.00E-24 |
| SH2D1A | CFD | 0.429 | 3.86E-18 |
| SH2D1A | STK17B | 0.674 | 6.72E-51 |
| SH2D1A | CCR7 | 0.733 | 4.03E-64 |
| SH2D1A | TRPV2 | 0.535 | 4.17E-29 |
| SH2D1A | VAV1 | 0.8 | 1.55E-84 |
| SH2D1A | PPP1R18 | 0.462 | 3.25E-21 |
| SH2D1A | BCL2A1 | 0.554 | 1.95E-31 |
| SH2D1A | BASP1 | 0.538 | 1.70E-29 |
| SH2D1A | C1orf54 | 0.576 | 2.08E-34 |
| SH2D1A | CCL13 | 0.462 | 3.26E-21 |
| SH2D1A | EGR2 | 0.464 | 2.20E-21 |
| SH2D1A | CXCR6 | 0.888 | 9.98E-128 |
| SH2D1A | RUNX3 | 0.872 | 2.61E-117 |
| SH2D1A | FAM49A | 0.413 | 7.21E-17 |
| SH2D1A | CD79B | 0.629 | 1.41E-42 |
| SH2D1A | LY86 | 0.624 | 8.68E-42 |
| SH2D1A | LSP1 | 0.732 | 6.90E-64 |
| SH2D1A | RHOG | 0.43 | 2.94E-18 |
| SH2D1A | IL4I1 | 0.587 | 4.60E-36 |
| SH2D1A | FCGR1A | 0.629 | 1.31E-42 |
| SH2D1A | P2RY8 | 0.413 | 7.06E-17 |
| SH2D1A | PTPN6 | 0.62 | 4.40E-41 |
| SH2D1A | EPSTI1 | 0.576 | 1.83E-34 |
| SH2D1A | FYB1 | 0.826 | 8.77E-95 |
| SH2D1A | TRPM2 | 0.469 | 8.15E-22 |
| SH2D1A | CD84 | 0.78 | 1.36E-77 |
| SH2D1A | C1QA | 0.655 | 2.77E-47 |
| SH2D1A | INPP4A | 0.416 | 4.24E-17 |
| SH2D1A | LTB | 0.522 | 1.45E-27 |
| SH2D1A | SH3BGRL | 0.462 | 3.94E-21 |
| SH2D1A | TNFRSF4 | 0.418 | 2.80E-17 |
| SH2D1A | PRKCH | 0.73 | 1.81E-63 |
| SH2D1A | FERMT3 | 0.764 | 7.27E-73 |
| SH2D1A | SPOCK2 | 0.767 | 9.47E-74 |
| SH2D1A | SLC38A5 | 0.443 | 2.29E-19 |
| SH2D1A | C1QB | 0.68 | 3.70E-52 |
| SH2D1A | IQGAP1 | 0.459 | 6.87E-21 |
| SH2D1A | CELF2 | 0.754 | 5.53E-70 |
| SH2D1A | MPEG1 | 0.726 | 2.12E-62 |
| SH2D1A | FCER1G | 0.643 | 5.43E-45 |
| SH2D1A | CCR1 | 0.624 | 9.03E-42 |
| SH2D1A | ZNF267 | 0.472 | 3.44E-22 |
| SH2D1A | UNC13D | 0.453 | 2.70E-20 |
| SH2D1A | MCUB | 0.463 | 3.16E-21 |
| SH2D1A | SIRPG | 0.908 | 6.27E-143 |
| SH2D1A | ARAP2 | 0.417 | 3.75E-17 |
| SH2D1A | IL18BP | 0.745 | 1.88E-67 |
| SH2D1A | LCK | 0.937 | 3.25E-172 |
| SH2D1A | IKBKE | 0.418 | 2.97E-17 |
| SH2D1A | ITM2A | 0.638 | 3.39E-44 |
| SH2D1A | SLC7A7 | 0.545 | 2.63E-30 |
| SH2D1A | RCSD1 | 0.758 | 6.59E-71 |
| SH2D1A | TBXAS1 | 0.558 | 6.24E-32 |
| SH2D1A | ZEB2 | 0.594 | 4.20E-37 |
| SH2D1A | COTL1 | 0.72 | 5.25E-61 |
| SH2D1A | CMTM7 | 0.443 | 1.95E-19 |
| SH2D1A | IL2RG | 0.708 | 2.95E-58 |
| SH2D1A | STAT4 | 0.442 | 2.63E-19 |
| SH2D1A | C1orf162 | 0.69 | 3.30E-54 |
| SH2D1A | SLC37A2 | 0.443 | 2.20E-19 |
| SH2D1A | TYROBP | 0.62 | 4.36E-41 |
| SH2D1A | RELT | 0.535 | 3.88E-29 |
| SH2D1A | PSMB9 | 0.548 | 9.87E-31 |
| SH2D1A | BTK | 0.796 | 4.57E-83 |
| SH2D1A | GPSM3 | 0.794 | 2.09E-82 |
| SH2D1A | HLA-DMB | 0.716 | 5.11E-60 |
| SH2D1A | EHBP1L1 | 0.403 | 5.09E-16 |
| SH2D1A | KLRB1 | 0.675 | 5.39E-51 |
| SH2D1A | DENND2D | 0.531 | 1.50E-28 |
| SH2D1A | GPR34 | 0.459 | 7.66E-21 |
| SH2D1A | IGSF6 | 0.725 | 3.36E-62 |
| SH2D1A | CTSC | 0.432 | 2.06E-18 |
| SH2D1A | FGL2 | 0.761 | 7.23E-72 |
| SH2D1A | CLEC2B | 0.624 | 8.94E-42 |
| SH2D1A | PPM1M | 0.455 | 1.64E-20 |
| SH2D1A | SLC7A8 | 0.434 | 1.22E-18 |
| SH2D1A | CNRIP1 | 0.415 | 5.78E-17 |
| SH2D1A | LILRB2 | 0.768 | 5.91E-74 |
| SH2D1A | CALHM2 | 0.418 | 3.28E-17 |
| SH2D1A | FCGR2A | 0.505 | 1.31E-25 |
| SH2D1A | BATF | 0.449 | 5.75E-20 |
| SH2D1A | CXCL10 | 0.586 | 8.01E-36 |
| SH2D1A | HLA-F | 0.408 | 1.98E-16 |
| SH2D1A | CD96 | 0.905 | 7.17E-140 |
| SH2D1A | APOBEC3C | 0.678 | 1.14E-51 |
| SH2D1A | HLA-DPB1 | 0.69 | 4.26E-54 |
| SH2D1A | VSIR | 0.507 | 8.28E-26 |
| SH2D1A | CLEC10A | 0.642 | 7.64E-45 |
| SH2D1A | AMPD3 | 0.439 | 4.31E-19 |
| SH2D1A | HLA-DQA1 | 0.748 | 2.61E-68 |
| SH2D1A | CXCL9 | 0.769 | 2.43E-74 |
| SH2D1A | LGALS9 | 0.478 | 8.88E-23 |
| SH2D1A | GIMAP7 | 0.644 | 3.24E-45 |
| SH2D1A | FOLR2 | 0.551 | 3.91E-31 |
| SH2D1A | EAF2 | 0.472 | 3.81E-22 |
| SH2D1A | FNBP1 | 0.455 | 1.76E-20 |
| SH2D1A | ZAP70 | 0.916 | 3.27E-149 |
| SH2D1A | STAC3 | 0.452 | 2.88E-20 |
| SH2D1A | EVI2A | 0.804 | 4.73E-86 |
| SH2D1A | SH3BGRL3 | 0.408 | 1.87E-16 |
| SH2D1A | FPR3 | 0.636 | 9.95E-44 |
| SH2D1A | PLEKHA2 | 0.49 | 6.16E-24 |
| SH2D1A | CSF1R | 0.628 | 2.02E-42 |
| SH2D1A | FCGR3A | 0.614 | 3.45E-40 |
| SH2D1A | SLAMF7 | 0.883 | 1.80E-124 |
| SH2D1A | IL10RA | 0.791 | 1.79E-81 |
| SH2D1A | CSF3R | 0.457 | 1.10E-20 |
| SH2D1A | ADA2 | 0.684 | 6.78E-53 |
| SH2D1A | HLA-DMA | 0.546 | 1.90E-30 |
| SH2D1A | DRAM1 | 0.442 | 2.67E-19 |
| SH2D1A | CST7 | 0.888 | 1.75E-127 |
| SH2D1A | CBLB | 0.405 | 3.31E-16 |
| SH2D1A | MNDA | 0.637 | 6.39E-44 |
| SH2D1A | CD5L | 0.456 | 1.24E-20 |
| SH2D1A | CD274 | 0.587 | 6.03E-36 |
| SH2D1A | NCF4 | 0.68 | 5.25E-52 |
| SH2D1A | CD300LF | 0.7 | 1.92E-56 |
| SH2D1A | MEF2C | 0.423 | 1.10E-17 |
| SH2D1A | CMKLR1 | 0.747 | 5.63E-68 |
| SH2D1A | EVL | 0.407 | 2.33E-16 |
| SH2D1A | HLA-A | 0.414 | 6.05E-17 |
| SH2D1A | KCTD12 | 0.541 | 8.75E-30 |
| SH2D1A | GZMA | 0.871 | 3.79E-117 |
| SH2D1A | CLEC4A | 0.654 | 5.44E-47 |
| SH2D1A | FGD3 | 0.63 | 1.01E-42 |
| SH2D1A | MYO1F | 0.79 | 3.90E-81 |
| SH2D1A | VIM | 0.442 | 2.42E-19 |
| SH2D1A | RASSF5 | 0.526 | 5.21E-28 |
| SH2D1A | C5AR1 | 0.414 | 6.92E-17 |
| SH2D1A | CCL24 | 0.435 | 1.04E-18 |
| SH2D1A | SLA2 | 0.936 | 1.15E-170 |
| SH2D1A | GPRIN3 | 0.406 | 2.99E-16 |
| SH2D1A | ETV7 | 0.554 | 1.73E-31 |
| SH2D1A | CASP4 | 0.404 | 3.75E-16 |
| SH2D1A | HLA-DRA | 0.681 | 3.50E-52 |
| SH2D1A | SP110 | 0.498 | 7.60E-25 |
| SH2D1A | SLFN11 | 0.473 | 2.91E-22 |
| SH2D1A | PPP1R16B | 0.603 | 1.82E-38 |
| SH2D1A | FLT3LG | 0.531 | 1.19E-28 |
| SH2D1A | GMIP | 0.546 | 2.20E-30 |
| SH2D1A | LAPTM5 | 0.687 | 1.85E-53 |
| SH2D1A | CYTH4 | 0.721 | 3.00E-61 |
| SH2D1A | CSK | 0.451 | 3.59E-20 |
| SH2D1A | CCL19 | 0.493 | 2.57E-24 |
| SH2D1A | CD72 | 0.824 | 8.27E-94 |
| SH2D1A | BATF2 | 0.527 | 3.82E-28 |
| SH2D1A | BCL2 | 0.461 | 4.54E-21 |
| SH2D1A | SAMHD1 | 0.468 | 9.05E-22 |
| SH2D1A | PLAU | 0.465 | 1.66E-21 |
| SH2D1A | PLA2G2D | 0.803 | 1.67E-85 |
| SH2D1A | TUBA1A | 0.403 | 4.50E-16 |
| SH2D1A | CD8B | 0.84 | 6.49E-101 |
| SH2D1A | AKNA | 0.721 | 3.03E-61 |
| SH2D1A | CD27 | 0.911 | 1.56E-145 |
| SH2D1A | ARHGAP30 | 0.839 | 2.82E-100 |
| SH2D1A | ST8SIA4 | 0.648 | 5.57E-46 |
| SH2D1A | ELF4 | 0.428 | 4.16E-18 |
| SH2D1A | LDHB | 0.498 | 8.48E-25 |
| SH2D1A | PREX1 | 0.543 | 4.68E-30 |
| SH2D1A | PLTP | 0.499 | 5.98E-25 |
| SH2D1A | HLA-DRB5 | 0.555 | 1.24E-31 |
| SH2D1A | NFKBID | 0.493 | 2.96E-24 |
| SH2D1A | GNB4 | 0.564 | 8.95E-33 |
| SH2D1A | AIF1 | 0.647 | 8.49E-46 |
| SH2D1A | CYTIP | 0.828 | 1.26E-95 |
| SH2D1A | STAT1 | 0.574 | 4.17E-34 |
| SH2D1A | UCP2 | 0.742 | 1.44E-66 |
| SH2D1A | SPN | 0.854 | 1.63E-107 |
| SH2D1A | SLC15A3 | 0.588 | 3.46E-36 |
| SH2D1A | SYTL1 | 0.607 | 5.40E-39 |
| SH2D1A | GPR183 | 0.583 | 1.88E-35 |
| SH2D1A | CSF2RA | 0.455 | 1.76E-20 |
| SH2D1A | CEACAM21 | 0.591 | 1.21E-36 |
| SH2D1A | ITGAX | 0.583 | 2.24E-35 |
| SH2D1A | CLEC2D | 0.814 | 7.42E-90 |
| SH2D1A | CD163L1 | 0.411 | 1.07E-16 |
| SH2D1A | APOBEC3G | 0.768 | 5.12E-74 |
| SH2D1A | SLAMF6 | 0.952 | 1.41E-193 |
| SH2D1A | IKZF1 | 0.882 | 1.35E-123 |
| SH2D1A | CD86 | 0.759 | 3.02E-71 |
| SH2D1A | CYLD | 0.432 | 2.09E-18 |
| SH2D1A | CD8A | 0.923 | 9.50E-157 |
| SH2D1A | PLEK | 0.764 | 9.76E-73 |
| SH2D1A | GNLY | 0.478 | 9.45E-23 |
| SH2D1A | DOK2 | 0.78 | 8.69E-78 |
| SH2D1A | LPXN | 0.738 | 1.87E-65 |
| SH2D1A | C19orf38 | 0.48 | 6.25E-23 |
| SH2D1A | RAC2 | 0.773 | 1.98E-75 |
| SH2D1A | ISG20 | 0.412 | 9.43E-17 |
| SH2D1A | HSH2D | 0.473 | 2.71E-22 |
| SH2D1A | CCR5 | 0.882 | 8.64E-124 |
| SH2D1A | PLEKHO2 | 0.61 | 1.75E-39 |
| SH2D1A | KCNK6 | 0.543 | 4.73E-30 |
| SH2D1A | TRAF1 | 0.525 | 7.13E-28 |
| SH2D1A | IRF1 | 0.608 | 3.76E-39 |
| SH2D1A | CHN1 | 0.408 | 1.85E-16 |
| SH2D1A | RAB8B | 0.446 | 1.03E-19 |
| SH2D1A | PCED1B | 0.614 | 4.00E-40 |
| SH2D1A | TMC8 | 0.815 | 3.16E-90 |
| SH2D1A | SH2D2A | 0.71 | 1.48E-58 |
| SH2D1A | ZNF101 | 0.518 | 4.82E-27 |
| SH2D1A | DOCK8 | 0.667 | 1.63E-49 |
| SH2D1A | ZNF385A | 0.436 | 9.47E-19 |
| SH2D1A | NOD1 | 0.402 | 6.12E-16 |
| SH2D1A | FPR1 | 0.591 | 1.47E-36 |
| SH2D1A | FUT8 | 0.492 | 3.65E-24 |
| SH2D1A | CCL5 | 0.843 | 3.36E-102 |
| SH2D1A | CALHM6 | 0.806 | 1.06E-86 |
| SH2D1A | CD163 | 0.6 | 7.20E-38 |
| SH2D1A | TAP1 | 0.575 | 2.57E-34 |
| SH2D1A | ARHGDIB | 0.75 | 1.25E-68 |
| SH2D1A | IRF8 | 0.536 | 2.96E-29 |
| SH2D1A | LCP2 | 0.822 | 3.75E-93 |
| SH2D1A | ACAP1 | 0.89 | 4.00E-129 |
| SH2D1A | NRROS | 0.696 | 2.33E-55 |
| SH2D1A | AXL | 0.548 | 9.94E-31 |
| SH2D1A | ARHGAP15 | 0.788 | 2.36E-80 |
| SH2D1A | FCMR | 0.838 | 8.20E-100 |
| SH2D1A | LILRB1 | 0.803 | 1.56E-85 |
| SH2D1A | CTSW | 0.793 | 4.20E-82 |
| SH2D1A | INPP5D | 0.756 | 2.18E-70 |
| SH2D1A | NFAM1 | 0.709 | 2.05E-58 |
| SH2D1A | DOK1 | 0.512 | 2.22E-26 |
| SH2D1A | GAL3ST4 | 0.421 | 1.61E-17 |
| SH2D1A | RNF166 | 0.53 | 1.80E-28 |
| SH2D1A | HCK | 0.728 | 7.16E-63 |
| SH2D1A | OTULINL | 0.525 | 7.96E-28 |
| SH2D1A | CD52 | 0.769 | 3.00E-74 |
| SH2D1A | NKG7 | 0.807 | 5.50E-87 |
| SH2D1A | STK10 | 0.645 | 2.59E-45 |
| SH2D1A | GBP4 | 0.671 | 3.42E-50 |
| SH2D1A | B2M | 0.493 | 2.74E-24 |
| SH2D1A | LRMP | 0.694 | 6.11E-55 |
| SH2D1A | TNFRSF1B | 0.534 | 5.24E-29 |
| SH2D1A | NABP1 | 0.409 | 1.76E-16 |
| SH2D1A | NLRC5 | 0.627 | 3.08E-42 |
| SH2D1A | CYBB | 0.706 | 1.01E-57 |
| SH2D1A | CTSS | 0.535 | 4.13E-29 |
| SH2D1A | MS4A7 | 0.673 | 1.19E-50 |
| SH2D1A | CD83 | 0.538 | 1.83E-29 |
| SH2D1A | WAS | 0.804 | 7.22E-86 |
| SH2D1A | DOCK10 | 0.662 | 1.96E-48 |
| SH2D1A | PLAC8 | 0.482 | 3.49E-23 |
| SH2D1A | MS4A6A | 0.684 | 7.95E-53 |
| SH2D1A | SLA | 0.832 | 3.95E-97 |
| SH2D1A | CD48 | 0.89 | 7.55E-129 |
| SH2D1A | GIMAP4 | 0.738 | 2.07E-65 |
| SH2D1A | EMB | 0.699 | 4.40E-56 |
| SH2D1A | SIGLEC1 | 0.678 | 1.26E-51 |
| SH2D1A | CARD16 | 0.612 | 7.52E-40 |
| SH2D1A | NCKAP1L | 0.826 | 9.82E-95 |
| SH2D1A | CCL2 | 0.402 | 5.74E-16 |
| SH2D1A | PDE4B | 0.418 | 2.97E-17 |
| SH2D1A | TBC1D10C | 0.708 | 4.04E-58 |
| SH2D1A | UBE2L6 | 0.43 | 2.67E-18 |
| SH2D1A | NFATC2 | 0.427 | 4.87E-18 |
| SH2D1A | CCL3 | 0.615 | 2.64E-40 |
| SH2D1A | GIMAP6 | 0.649 | 3.74E-46 |
| SH2D1A | CERKL | 0.761 | 8.86E-72 |
| SH2D1A | CCND2 | 0.562 | 1.55E-32 |
| SH2D1A | TNFRSF18 | 0.47 | 5.58E-22 |
| SH2D1A | LYL1 | 0.571 | 9.32E-34 |
| SH2D1A | VCAM1 | 0.438 | 5.37E-19 |
| SH2D1A | CDC42EP3 | 0.432 | 1.78E-18 |
| SH2D1A | OSCAR | 0.521 | 2.24E-27 |
| SH2D1A | IL18 | 0.568 | 2.45E-33 |
| SH2D1A | AP1S2 | 0.499 | 6.19E-25 |
| SH2D1A | MOXD1 | 0.406 | 2.63E-16 |
| SH2D1A | CD3E | 0.913 | 4.28E-147 |
| SH2D1A | HLA-DRB1 | 0.629 | 1.17E-42 |
| SH2D1A | PTPRC | 0.859 | 3.10E-110 |
| SH2D1A | MMP19 | 0.401 | 7.43E-16 |
| SH2D1A | LILRB4 | 0.697 | 1.24E-55 |
| SH2D1A | C1QC | 0.688 | 9.78E-54 |
| SH2D1A | FCHSD1 | 0.404 | 4.24E-16 |
| SH2D1A | CD5 | 0.9 | 1.94E-136 |
| SH2D1A | LGALS2 | 0.56 | 2.93E-32 |
| SH2D1A | CD1C | 0.473 | 2.80E-22 |
| SH2D1A | HAPLN3 | 0.755 | 2.77E-70 |
| SH2D1A | CD7 | 0.546 | 1.88E-30 |
| SH2D1A | ANKRD44 | 0.597 | 1.81E-37 |
| SH2D1A | TOX | 0.414 | 6.22E-17 |
| SH2D1A | CD2 | 0.878 | 2.83E-121 |
| SH2D1A | TMEM243 | 0.46 | 6.15E-21 |
| SH2D1A | CORO1A | 0.841 | 1.99E-101 |
| SH2D1A | WARS | 0.681 | 2.85E-52 |
| SH2D1A | IL16 | 0.878 | 6.71E-121 |
| SH2D1A | FGR | 0.619 | 5.38E-41 |
| SH2D1A | EPB41L3 | 0.652 | 1.02E-46 |
| SH2D1A | APOBEC3D | 0.589 | 2.41E-36 |
| SH2D1A | MZB1 | 0.738 | 1.48E-65 |
| SH2D1A | CXCR4 | 0.614 | 3.91E-40 |
| SH2D1A | CARD11 | 0.603 | 2.34E-38 |
| SH2D1A | HLA-DQB2 | 0.568 | 2.51E-33 |
| SH2D1A | SYK | 0.615 | 2.78E-40 |
| SH2D1A | CXCL13 | 0.52 | 2.63E-27 |
| SH2D1A | PIK3R5 | 0.768 | 5.64E-74 |
| SH2D1A | EVI2B | 0.838 | 4.50E-100 |
| SH2D1A | PTAFR | 0.561 | 2.10E-32 |
| SH2D1A | LXN | 0.436 | 8.47E-19 |
| SH2D1A | CXCL11 | 0.559 | 3.87E-32 |
| SH2D1A | TLR4 | 0.441 | 3.34E-19 |
| SH2D1A | ATP2A3 | 0.712 | 3.54E-59 |
| SH2D1A | SNX20 | 0.881 | 5.18E-123 |
| SH2D1A | PSMB10 | 0.522 | 1.74E-27 |
| SH2D1A | DOCK2 | 0.839 | 1.58E-100 |
| SH2D1A | RGS19 | 0.408 | 1.89E-16 |
| SH2D1A | HOXB2 | 0.46 | 5.12E-21 |
| SH2D1A | DGKZ | 0.452 | 3.26E-20 |
| SH2D1A | AOAH | 0.766 | 2.75E-73 |
| SH2D1A | TRAFD1 | 0.477 | 1.26E-22 |
| SH2D1A | ANXA1 | 0.503 | 2.42E-25 |
| SH2D1A | CLIC2 | 0.538 | 1.66E-29 |
| SH2D1A | IL7R | 0.682 | 2.03E-52 |
| SH2D1A | TRAF5 | 0.402 | 6.01E-16 |
| SH2D1A | ABI3 | 0.703 | 6.44E-57 |
| SH2D1A | ADGRE5 | 0.457 | 9.82E-21 |
| SH2D1A | ADAM19 | 0.449 | 6.20E-20 |
| SH2D1A | SH2D1A | 1 | 0 |
| SH2D1A | CFP | 0.566 | 4.19E-33 |
| SH2D1A | LILRB5 | 0.469 | 7.50E-22 |
| SH2D1A | RILPL2 | 0.534 | 5.88E-29 |
| SH2D1A | ENTPD1 | 0.491 | 4.19E-24 |
| SH2D1A | HAVCR2 | 0.649 | 4.22E-46 |
| SH2D1A | HCST | 0.69 | 3.17E-54 |
| SH2D1A | GLIPR1 | 0.441 | 2.91E-19 |
| SH2D1A | TAGAP | 0.846 | 9.85E-104 |
| SH2D1A | CTLA4 | 0.821 | 1.89E-92 |
| SH2D1A | HLA-DOB | 0.627 | 3.47E-42 |
| SH2D1A | MICAL1 | 0.546 | 1.77E-30 |
| SH2D1A | MATK | 0.451 | 4.30E-20 |
| SH2D1A | MILR1 | 0.625 | 5.80E-42 |
| SH2D1A | HVCN1 | 0.751 | 5.39E-69 |
| SH2D1A | RNASE6 | 0.624 | 1.13E-41 |
| SH2D1A | GNGT2 | 0.754 | 6.16E-70 |
| SH2D1A | GBGT1 | 0.41 | 1.24E-16 |
| SH2D1A | ARHGAP45 | 0.534 | 6.22E-29 |
| SH2D1A | APOBR | 0.585 | 1.15E-35 |
| SH2D1A | ARHGAP31 | 0.412 | 8.71E-17 |
| SH2D1A | PDE6G | 0.732 | 4.54E-64 |
| SH2D1A | JAK3 | 0.783 | 1.05E-78 |
| SH2D1A | P2RY13 | 0.713 | 2.56E-59 |
| SH2D1A | XCL2 | 0.635 | 1.31E-43 |
| SH2D1A | LGMN | 0.577 | 1.27E-34 |
| SH2D1A | NMI | 0.41 | 1.35E-16 |
| SH2D1A | STK4 | 0.434 | 1.33E-18 |
| SH2D1A | MYO1G | 0.846 | 7.88E-104 |
| SH2D1A | FAM78A | 0.796 | 5.12E-83 |
| SH2D1A | PIM2 | 0.653 | 7.78E-47 |
| SH2D1A | ARHGAP4 | 0.401 | 6.59E-16 |
| SH2D1A | RAP2B | 0.42 | 1.86E-17 |
| SH2D1A | CHST2 | 0.587 | 4.60E-36 |
| SH2D1A | IGLL5 | 0.75 | 6.93E-69 |
| SH2D1A | ALOX5AP | 0.578 | 9.17E-35 |
| SH2D1A | TENT5C | 0.406 | 2.87E-16 |
| SH2D1A | TNFAIP8 | 0.595 | 4.02E-37 |
| SH2D1A | MAP4K1 | 0.928 | 1.73E-161 |
| SH2D1A | FLI1 | 0.612 | 8.69E-40 |
| SH2D1A | HLA-DOA | 0.75 | 9.36E-69 |
| SH2D1A | CCL8 | 0.6 | 6.16E-38 |
| SH2D1A | FMNL1 | 0.668 | 1.16E-49 |
| SH2D1A | RAB31 | 0.496 | 1.23E-24 |
| SH2D1A | SELPLG | 0.798 | 7.98E-84 |
| SH2D1A | STAT5A | 0.518 | 4.67E-27 |
| SH2D1A | DENND3 | 0.457 | 1.14E-20 |
| SH2D1A | DOK3 | 0.709 | 1.75E-58 |
| SH2D1A | ITGAL | 0.558 | 4.83E-32 |
| SH2D1A | IDO1 | 0.653 | 9.02E-47 |
| SH2D1A | TRIM22 | 0.51 | 3.87E-26 |
| SH2D1A | PML | 0.403 | 4.84E-16 |
| SH2D1A | CCL4L2 | 0.611 | 1.41E-39 |
| SH2D1A | PARVG | 0.811 | 1.24E-88 |
| SH2D1A | PLAUR | 0.45 | 4.94E-20 |
| SH2D1A | S100A4 | 0.55 | 5.85E-31 |
| SH2D1A | C3AR1 | 0.621 | 2.83E-41 |
| SH2D1A | MOB3A | 0.427 | 5.77E-18 |
| SH2D1A | TNFAIP8L2 | 0.695 | 3.62E-55 |
| SH2D1A | NECAP2 | 0.483 | 2.83E-23 |
| SH2D1A | SIGLEC9 | 0.612 | 9.61E-40 |
| SH2D1A | MMP9 | 0.497 | 9.31E-25 |
| SH2D1A | C16orf54 | 0.878 | 5.67E-121 |
| SH2D1A | SAMSN1 | 0.813 | 1.92E-89 |
| SH2D1A | DUSP2 | 0.76 | 1.16E-71 |
| SH2D1A | ARHGAP18 | 0.42 | 2.19E-17 |
| SH2D1A | SASH3 | 0.872 | 3.29E-117 |
| SH2D1A | FXYD5 | 0.406 | 3.10E-16 |
| SH2D1A | HLA-DQA2 | 0.491 | 4.23E-24 |
| SH2D1A | MSR1 | 0.504 | 1.61E-25 |
| SH2D1A | RGS1 | 0.607 | 4.94E-39 |
| SH2D1A | CD53 | 0.808 | 2.43E-87 |
| SH2D1A | ADAM8 | 0.461 | 4.21E-21 |
| SH2D1A | GZMM | 0.726 | 2.28E-62 |
| SH2D1A | SGTB | 0.407 | 2.41E-16 |
| SH2D1A | SEMA4A | 0.678 | 1.14E-51 |
| SH2D1A | RASAL3 | 0.846 | 9.33E-104 |
| SH2D1A | CD3D | 0.77 | 1.90E-74 |
| SH2D1A | STX11 | 0.579 | 7.76E-35 |
| SH2D1A | MARCO | 0.404 | 3.77E-16 |
| SH2D1A | CMTM3 | 0.402 | 5.75E-16 |
| SH2D1A | NRP2 | 0.422 | 1.51E-17 |
| SH2D1A | GIMAP1 | 0.677 | 1.91E-51 |
| SH2D1A | SPI1 | 0.692 | 1.70E-54 |
| SH2D1A | FAM129A | 0.459 | 6.30E-21 |
| SH2D1A | FCHO1 | 0.401 | 6.83E-16 |
| SH2D1A | CD209 | 0.5 | 4.72E-25 |
| SH2D1A | DGKA | 0.673 | 1.15E-50 |
| SH2D1A | LAG3 | 0.761 | 8.33E-72 |
| SH2D1A | HLA-DPA1 | 0.666 | 2.74E-49 |
| SH2D1A | LYN | 0.416 | 4.74E-17 |
| SH2D1A | CD6 | 0.901 | 8.84E-137 |
| SH2D1A | CD69 | 0.812 | 6.41E-89 |
| SH2D1A | HK1 | 0.405 | 3.20E-16 |
| SH2D1A | TMEM229B | 0.532 | 1.05E-28 |
| SH2D1A | PDCD1 | 0.711 | 7.81E-59 |
| SH2D1A | SIGLEC7 | 0.711 | 8.42E-59 |
| SH2D1A | SLAMF8 | 0.755 | 4.53E-70 |
| SH2D1A | ARHGEF1 | 0.438 | 6.36E-19 |
| SH2D1A | RAB42 | 0.471 | 4.33E-22 |
| SH2D1A | SLC2A3 | 0.414 | 6.75E-17 |
| SH2D1A | LILRA5 | 0.582 | 2.48E-35 |
| SH2D1A | RARRES3 | 0.446 | 1.14E-19 |
| SH2D1A | SEMA4D | 0.742 | 1.86E-66 |
| SH2D1A | IKZF3 | 0.927 | 1.02E-160 |
| SH2D1A | VSIG4 | 0.466 | 1.46E-21 |
| SH2D1A | SEPT1 | 0.783 | 9.06E-79 |
| SH2D1A | MOB3C | 0.417 | 3.30E-17 |
| SH2D1A | GAS7 | 0.455 | 1.61E-20 |
| SH2D1A | CLEC7A | 0.681 | 2.72E-52 |
| SH2D1A | LIMD2 | 0.611 | 1.17E-39 |
| SH2D1A | BTN3A2 | 0.407 | 2.36E-16 |
| SH2D1A | CD300C | 0.597 | 1.78E-37 |
| SH2D1A | IL12RB1 | 0.862 | 9.22E-112 |
| SH2D1A | PDE7A | 0.444 | 1.55E-19 |
| SH2D1A | REC8 | 0.525 | 7.06E-28 |
| SH2D1A | CCDC88B | 0.599 | 7.55E-38 |
| SH2D1A | SRGN | 0.693 | 7.81E-55 |
| SH2D1A | RASSF2 | 0.635 | 1.56E-43 |
| SH2D1A | C21orf91 | 0.456 | 1.32E-20 |
| SH2D1A | KCNN4 | 0.617 | 1.57E-40 |
| SH2D1A | GZMH | 0.767 | 1.48E-73 |
| SH2D1A | LPAR6 | 0.52 | 2.51E-27 |
| SH2D1A | CD4 | 0.612 | 8.27E-40 |
| SH2D1A | IL2RB | 0.86 | 1.61E-110 |
| SH2D1A | HPSE | 0.569 | 1.99E-33 |
| SH2D1A | MLKL | 0.44 | 3.75E-19 |
| SH2D1A | MFNG | 0.534 | 6.55E-29 |
| SH2D1A | GZMK | 0.929 | 1.54E-162 |
| SH2D1A | BTN3A3 | 0.407 | 2.30E-16 |
| SH2D1A | ETS1 | 0.524 | 8.48E-28 |
| SH2D1A | OSTF1 | 0.447 | 9.83E-20 |
| SH2D1A | PLCB2 | 0.781 | 4.54E-78 |
| SH2D1A | GPNMB | 0.583 | 1.90E-35 |
| SH2D1A | LCP1 | 0.671 | 2.45E-50 |
| SH2D1A | DOCK11 | 0.589 | 2.64E-36 |
| SH2D1A | CD300A | 0.628 | 2.14E-42 |
| SH2D1A | TIMD4 | 0.54 | 9.57E-30 |
| SH2D1A | SCIMP | 0.814 | 1.15E-89 |
| SH2D1A | SDC3 | 0.553 | 2.67E-31 |
| SH2D1A | GZMB | 0.738 | 2.07E-65 |
| SH2D1A | CDC42SE2 | 0.408 | 2.09E-16 |
| SH2D1A | CD79A | 0.779 | 1.63E-77 |
| SH2D1A | SIT1 | 0.923 | 1.55E-156 |
| SH2D1A | SLC9A9 | 0.627 | 3.03E-42 |
| SH2D1A | CASP1 | 0.655 | 3.83E-47 |
| SH2D1A | MS4A4A | 0.642 | 9.27E-45 |
| SH2D1A | CCL18 | 0.532 | 1.03E-28 |
| SH2D1A | LOXL1 | 0.401 | 7.60E-16 |
| SH2D1A | CD74 | 0.621 | 2.71E-41 |
| SH2D1A | PIK3CD | 0.861 | 2.25E-111 |
| SH2D1A | CCL4 | 0.807 | 3.63E-87 |
| SH2D1A | DEF6 | 0.743 | 6.22E-67 |
| SH2D1A | PTGER4 | 0.633 | 3.21E-43 |
| SH2D1A | CGAS | 0.431 | 2.20E-18 |
| SH2D1A | ARHGAP9 | 0.881 | 5.28E-123 |
| SH2D1A | MSL3 | 0.443 | 2.04E-19 |
| SH2D1A | PLEKHO1 | 0.63 | 1.00E-42 |
| SH2D1A | JCHAIN | 0.795 | 1.07E-82 |
| SH2D1A | TMSB4X | 0.519 | 3.27E-27 |
| SH2D1A | HLA-E | 0.503 | 2.25E-25 |
| SH2D1A | ARHGEF6 | 0.593 | 6.20E-37 |
| SH2D1A | TMEM173 | 0.527 | 3.71E-28 |
| SH2D1A | XCL1 | 0.417 | 3.87E-17 |
| SH2D1A | GMFG | 0.674 | 7.04E-51 |
| SH2D1A | FCN1 | 0.686 | 2.03E-53 |
| SH2D1A | GCNT1 | 0.414 | 6.05E-17 |
| SH2D1A | THEMIS2 | 0.741 | 2.16E-66 |
| SH2D1A | CCR2 | 0.759 | 2.31E-71 |
| SH2D1A | CXCR3 | 0.881 | 8.56E-123 |
| SH2D1A | ARL4C | 0.535 | 4.68E-29 |
| SH2D1A | PTK2B | 0.463 | 2.86E-21 |
| SH2D1A | CIITA | 0.707 | 5.51E-58 |
| SH2D1A | PRF1 | 0.865 | 2.46E-113 |
| SH2D1A | LRRC25 | 0.553 | 2.73E-31 |
| SH2D1A | EBI3 | 0.707 | 7.12E-58 |
| SH2D1A | PARP8 | 0.466 | 1.53E-21 |
| SH2D1A | WIPF1 | 0.752 | 2.22E-69 |
| SH2D1A | CPVL | 0.476 | 1.39E-22 |
| SH2D1A | JAML | 0.525 | 6.67E-28 |
| SH2D1A | SH3BP1 | 0.497 | 9.84E-25 |
| SH2D1A | ITPKB | 0.525 | 7.91E-28 |
| SH2D1A | PRDM1 | 0.766 | 1.70E-73 |
| SH2D1A | CCRL2 | 0.485 | 1.88E-23 |
| SH2D1A | SYT11 | 0.522 | 1.70E-27 |
| SH2D1A | GFI1 | 0.443 | 2.30E-19 |
| SH2D1A | HLA-DQB1 | 0.673 | 9.30E-51 |
| SH2D1A | P2RY6 | 0.476 | 1.55E-22 |
| SH2D1A | CD247 | 0.919 | 2.40E-152 |

Table S2. The functions of SH2D1A co-expressed genes.

| Type | Description | P value |
| --- | --- | --- |
| BP | T cell activation | 3.36E-65 |
| BP | regulation of lymphocyte activation | 5.80E-60 |
| BP | regulation of T cell activation | 1.69E-52 |
| BP | leukocyte cell-cell adhesion | 3.67E-52 |
| BP | positive regulation of cell activation | 9.87E-52 |
| BP | regulation of leukocyte proliferation | 3.45E-50 |
| BP | positive regulation of leukocyte activation | 6.37E-50 |
| BP | leukocyte proliferation | 7.93E-50 |
| BP | regulation of lymphocyte proliferation | 8.79E-50 |
| BP | regulation of mononuclear cell proliferation | 1.24E-49 |
| BP | lymphocyte proliferation | 1.71E-49 |
| BP | mononuclear cell proliferation | 3.00E-49 |
| BP | positive regulation of lymphocyte activation | 4.83E-47 |
| BP | regulation of leukocyte cell-cell adhesion | 6.20E-47 |
| BP | positive regulation of leukocyte cell-cell adhesion | 6.78E-46 |
| BP | lymphocyte differentiation | 3.48E-45 |
| BP | positive regulation of T cell activation | 1.67E-44 |
| BP | regulation of cell-cell adhesion | 8.98E-44 |
| BP | positive regulation of cell-cell adhesion | 1.50E-43 |
| BP | positive regulation of cell adhesion | 6.97E-40 |
| BP | T cell proliferation | 1.35E-38 |
| BP | leukocyte migration | 1.28E-37 |
| BP | cellular response to interferon-gamma | 9.07E-37 |
| BP | positive regulation of lymphocyte proliferation | 1.34E-35 |
| BP | positive regulation of mononuclear cell proliferation | 1.96E-35 |
| BP | positive regulation of leukocyte proliferation | 2.09E-35 |
| BP | regulation of T cell proliferation | 2.60E-35 |
| BP | immune response-activating cell surface receptor signaling pathway | 6.42E-35 |
| BP | response to interferon-gamma | 2.13E-34 |
| BP | T cell differentiation | 2.83E-34 |
| BP | regulation of leukocyte differentiation | 2.87E-31 |
| BP | leukocyte chemotaxis | 1.22E-30 |
| BP | cell chemotaxis | 1.49E-30 |
| BP | regulation of lymphocyte differentiation | 4.15E-30 |
| BP | positive regulation of T cell proliferation | 5.20E-30 |
| BP | neutrophil activation | 3.03E-29 |
| BP | negative regulation of immune system process | 9.13E-29 |
| BP | antigen receptor-mediated signaling pathway | 1.04E-28 |
| BP | negative regulation of leukocyte activation | 2.58E-28 |
| BP | alpha-beta T cell activation | 2.78E-28 |
| BP | positive regulation of cytokine production | 6.75E-28 |
| BP | neutrophil degranulation | 1.49E-27 |
| BP | neutrophil activation involved in immune response | 2.16E-27 |
| BP | neutrophil mediated immunity | 8.13E-27 |
| BP | negative regulation of cell activation | 7.27E-26 |
| BP | regulation of hemopoiesis | 1.01E-25 |
| BP | myeloid leukocyte migration | 7.29E-25 |
| BP | granulocyte migration | 1.67E-24 |
| BP | regulation of immune effector process | 2.87E-24 |
| BP | adaptive immune response based on somatic recombination of immune receptors built from immunoglobulin superfamily domains | 5.31E-24 |
| BP | negative regulation of lymphocyte activation | 6.18E-24 |
| BP | regulation of T cell differentiation | 1.24E-23 |
| BP | interferon-gamma-mediated signaling pathway | 5.53E-23 |
| BP | neutrophil migration | 8.54E-23 |
| BP | T cell receptor signaling pathway | 1.25E-22 |
| BP | antigen processing and presentation | 1.39E-22 |
| BP | neutrophil chemotaxis | 2.61E-22 |
| BP | granulocyte chemotaxis | 3.48E-22 |
| BP | regulation of leukocyte migration | 3.59E-22 |
| BP | regulation of leukocyte mediated immunity | 9.38E-22 |
| BP | regulation of alpha-beta T cell activation | 1.80E-21 |
| BP | positive regulation of lymphocyte differentiation | 2.50E-21 |
| BP | positive regulation of leukocyte differentiation | 5.84E-21 |
| BP | positive regulation of immune effector process | 1.41E-20 |
| BP | regulation of leukocyte apoptotic process | 1.61E-20 |
| BP | positive regulation of response to external stimulus | 1.71E-20 |
| BP | lymphocyte mediated immunity | 1.76E-20 |
| BP | B cell activation | 1.97E-20 |
| BP | phagocytosis | 2.31E-20 |
| BP | negative regulation of leukocyte proliferation | 4.69E-20 |
| BP | leukocyte mediated cytotoxicity | 1.22E-19 |
| BP | positive regulation of leukocyte migration | 1.83E-19 |
| BP | regulation of innate immune response | 2.30E-19 |
| BP | positive regulation of hemopoiesis | 2.87E-19 |
| BP | regulation of B cell proliferation | 4.42E-19 |
| BP | negative regulation of cytokine production | 8.26E-19 |
| BP | cell killing | 9.29E-19 |
| BP | chemokine-mediated signaling pathway | 1.27E-18 |
| BP | response to chemokine | 1.29E-18 |
| BP | cellular response to chemokine | 1.29E-18 |
| BP | negative regulation of mononuclear cell proliferation | 2.31E-18 |
| BP | negative regulation of lymphocyte proliferation | 2.31E-18 |
| BP | regulation of tumor necrosis factor superfamily cytokine production | 3.21E-18 |
| BP | lymphocyte migration | 4.32E-18 |
| BP | response to molecule of bacterial origin | 7.17E-18 |
| BP | tumor necrosis factor superfamily cytokine production | 8.22E-18 |
| BP | leukocyte apoptotic process | 8.66E-18 |
| BP | response to lipopolysaccharide | 9.11E-18 |
| BP | antigen processing and presentation of exogenous antigen | 1.19E-17 |
| BP | positive regulation of alpha-beta T cell activation | 1.28E-17 |
| BP | regulation of adaptive immune response | 1.62E-17 |
| BP | negative regulation of immune response | 2.14E-17 |
| BP | antigen processing and presentation of exogenous peptide antigen | 2.89E-17 |
| BP | mononuclear cell migration | 3.17E-17 |
| BP | antigen processing and presentation of peptide antigen | 3.89E-17 |
| BP | alpha-beta T cell differentiation | 4.83E-17 |
| BP | positive regulation of T cell differentiation | 5.72E-17 |
| BP | negative regulation of T cell activation | 6.29E-17 |
| BP | interferon-gamma production | 7.95E-17 |
| BP | lymphocyte activation involved in immune response | 8.08E-17 |
| BP | positive regulation of cytosolic calcium ion concentration | 8.29E-17 |
| BP | alpha-beta T cell proliferation | 1.00E-16 |
| BP | regulation of leukocyte chemotaxis | 1.00E-16 |
| BP | B cell proliferation | 1.24E-16 |
| BP | positive regulation of GTPase activity | 1.77E-16 |
| BP | positive regulation of leukocyte chemotaxis | 1.80E-16 |
| BP | positive regulation of tumor necrosis factor superfamily cytokine production | 2.38E-16 |
| BP | lymphocyte chemotaxis | 4.64E-16 |
| BP | homeostasis of number of cells | 4.90E-16 |
| BP | regulation of interferon-gamma production | 5.65E-16 |
| BP | monocyte chemotaxis | 6.53E-16 |
| BP | regulation of adaptive immune response based on somatic recombination of immune receptors built from immunoglobulin superfamily domains | 6.64E-16 |
| BP | cellular response to biotic stimulus | 8.30E-16 |
| BP | regulation of B cell activation | 1.01E-15 |
| BP | regulation of tumor necrosis factor production | 1.17E-15 |
| BP | tumor necrosis factor production | 1.95E-15 |
| BP | negative regulation of leukocyte cell-cell adhesion | 2.48E-15 |
| BP | regulation of chemotaxis | 2.67E-15 |
| BP | regulation of cell killing | 3.09E-15 |
| BP | cellular response to lipopolysaccharide | 3.32E-15 |
| BP | interleukin-2 production | 3.81E-15 |
| BP | T cell selection | 4.59E-15 |
| BP | regulation of cytosolic calcium ion concentration | 4.76E-15 |
| BP | positive regulation of chemotaxis | 7.85E-15 |
| BP | CD4-positive, alpha-beta T cell activation | 8.16E-15 |
| BP | cellular response to molecule of bacterial origin | 8.84E-15 |
| BP | myeloid cell differentiation | 1.10E-14 |
| BP | regulation of lymphocyte mediated immunity | 1.15E-14 |
| BP | regulation of alpha-beta T cell proliferation | 1.70E-14 |
| BP | T cell mediated immunity | 1.91E-14 |
| BP | cellular calcium ion homeostasis | 2.02E-14 |
| BP | positive regulation of tumor necrosis factor production | 2.13E-14 |
| BP | regulation of GTPase activity | 2.61E-14 |
| BP | regulation of interleukin-10 production | 3.23E-14 |
| BP | B cell differentiation | 3.24E-14 |
| BP | negative regulation of cell-cell adhesion | 3.35E-14 |
| BP | regulation of inflammatory response | 4.18E-14 |
| BP | cytokine secretion | 5.25E-14 |
| BP | calcium ion homeostasis | 5.75E-14 |
| BP | cytokine biosynthetic process | 6.35E-14 |
| BP | cytokine metabolic process | 7.67E-14 |
| BP | interleukin-10 production | 9.27E-14 |
| BP | regulation of cell shape | 1.08E-13 |
| BP | T cell costimulation | 1.30E-13 |
| BP | CD4-positive, alpha-beta T cell differentiation | 1.39E-13 |
| BP | T cell migration | 1.46E-13 |
| BP | lymphocyte costimulation | 1.80E-13 |
| BP | T cell activation involved in immune response | 1.88E-13 |
| BP | negative regulation of leukocyte mediated immunity | 1.89E-13 |
| BP | positive regulation of innate immune response | 2.10E-13 |
| BP | leukocyte homeostasis | 2.43E-13 |
| BP | positive regulation of cytokine biosynthetic process | 2.61E-13 |
| BP | cellular divalent inorganic cation homeostasis | 3.09E-13 |
| BP | positive regulation of cell killing | 3.46E-13 |
| BP | regulation of leukocyte mediated cytotoxicity | 3.93E-13 |
| BP | negative regulation of cell adhesion | 4.13E-13 |
| BP | positive regulation of alpha-beta T cell proliferation | 5.14E-13 |
| BP | cellular defense response | 7.56E-13 |
| BP | myeloid leukocyte differentiation | 7.79E-13 |
| BP | regulation of cytokine biosynthetic process | 9.01E-13 |
| BP | regulation of interleukin-2 production | 1.05E-12 |
| BP | regulation of ERK1 and ERK2 cascade | 1.24E-12 |
| BP | ERK1 and ERK2 cascade | 1.31E-12 |
| BP | natural killer cell mediated immunity | 1.48E-12 |
| BP | negative regulation of leukocyte apoptotic process | 1.59E-12 |
| BP | regulation of cytokine secretion | 1.64E-12 |
| BP | dendritic cell migration | 2.73E-12 |
| BP | positive regulation of ERK1 and ERK2 cascade | 2.99E-12 |
| BP | positive T cell selection | 3.45E-12 |
| BP | negative regulation of T cell proliferation | 4.77E-12 |
| BP | positive regulation of adaptive immune response based on somatic recombination of immune receptors built from immunoglobulin superfamily domains | 4.93E-12 |
| BP | natural killer cell mediated cytotoxicity | 6.34E-12 |
| BP | regulation of lymphocyte apoptotic process | 1.13E-11 |
| BP | B cell receptor signaling pathway | 1.21E-11 |
| BP | positive regulation of lymphocyte migration | 1.22E-11 |
| BP | positive regulation of adaptive immune response | 1.27E-11 |
| BP | response to virus | 1.36E-11 |
| BP | regulation of alpha-beta T cell differentiation | 1.44E-11 |
| BP | dendritic cell chemotaxis | 1.49E-11 |
| BP | regulation of interleukin-12 production | 1.52E-11 |
| BP | regulation of leukocyte degranulation | 1.84E-11 |
| BP | regulation of myeloid leukocyte mediated immunity | 2.04E-11 |
| BP | positive regulation of interferon-gamma production | 2.41E-11 |
| BP | positive regulation of alpha-beta T cell differentiation | 2.56E-11 |
| BP | interleukin-12 production | 2.72E-11 |
| BP | antigen processing and presentation of exogenous peptide antigen via MHC class II | 3.00E-11 |
| BP | regulation of phagocytosis | 3.00E-11 |
| BP | regulation of T cell apoptotic process | 4.78E-11 |
| BP | T cell mediated cytotoxicity | 4.80E-11 |
| BP | T cell differentiation involved in immune response | 5.06E-11 |
| BP | antigen processing and presentation of peptide antigen via MHC class II | 5.22E-11 |
| BP | positive regulation of B cell proliferation | 5.46E-11 |
| BP | cellular response to tumor necrosis factor | 5.61E-11 |
| BP | positive regulation of inflammatory response | 5.94E-11 |
| BP | antigen processing and presentation of peptide or polysaccharide antigen via MHC class II | 6.25E-11 |
| BP | interleukin-1 production | 7.21E-11 |
| BP | regulation of interleukin-1 production | 7.46E-11 |
| BP | regulation of T cell mediated immunity | 8.10E-11 |
| BP | regulation of CD4-positive, alpha-beta T cell activation | 1.04E-10 |
| BP | T-helper 1 type immune response | 1.08E-10 |
| BP | T cell chemotaxis | 1.16E-10 |
| BP | regulation of B cell differentiation | 1.16E-10 |
| BP | positive regulation of leukocyte apoptotic process | 1.16E-10 |
| BP | positive regulation of leukocyte mediated immunity | 1.59E-10 |
| BP | negative regulation of immune effector process | 1.60E-10 |
| BP | regulation of lymphocyte migration | 1.70E-10 |
| BP | calcium ion transport | 2.05E-10 |
| BP | natural killer cell activation | 2.40E-10 |
| BP | CD8-positive, alpha-beta T cell activation | 2.47E-10 |
| BP | positive regulation of interleukin-2 production | 2.80E-10 |
| BP | response to tumor necrosis factor | 3.40E-10 |
| BP | positive regulation of leukocyte mediated cytotoxicity | 3.44E-10 |
| BP | interleukin-1 beta production | 4.27E-10 |
| BP | regulation of interleukin-1 beta production | 5.08E-10 |
| BP | cytosolic calcium ion transport | 5.79E-10 |
| BP | humoral immune response | 6.32E-10 |
| BP | calcium-mediated signaling | 6.34E-10 |
| BP | mast cell mediated immunity | 6.61E-10 |
| BP | eosinophil migration | 6.71E-10 |
| BP | lymphocyte apoptotic process | 6.75E-10 |
| BP | calcium ion transport into cytosol | 7.02E-10 |
| BP | positive regulation of interleukin-1 production | 7.29E-10 |
| BP | negative regulation of innate immune response | 7.29E-10 |
| BP | regulation of calcium ion transport | 8.60E-10 |
| BP | regulation of T cell mediated cytotoxicity | 9.19E-10 |
| BP | CD4-positive, alpha-beta T cell differentiation involved in immune response | 9.27E-10 |
| BP | mast cell activation | 9.27E-10 |
| BP | peptidyl-tyrosine phosphorylation | 1.05E-09 |
| BP | alpha-beta T cell activation involved in immune response | 1.17E-09 |
| BP | alpha-beta T cell differentiation involved in immune response | 1.17E-09 |
| BP | cellular extravasation | 1.17E-09 |
| BP | actin polymerization or depolymerization | 1.28E-09 |
| BP | peptidyl-tyrosine modification | 1.30E-09 |
| BP | regulation of antigen processing and presentation | 1.38E-09 |
| BP | regulation of cytokine production involved in immune response | 1.45E-09 |
| BP | positive regulation of interleukin-1 beta production | 1.48E-09 |
| BP | regulation of lymphocyte chemotaxis | 1.65E-09 |
| BP | positive regulation of interleukin-12 production | 1.88E-09 |
| BP | regulation of CD8-positive, alpha-beta T cell activation | 2.10E-09 |
| BP | regulation of production of molecular mediator of immune response | 2.34E-09 |
| BP | thymic T cell selection | 2.35E-09 |
| BP | activation of innate immune response | 2.41E-09 |
| BP | positive regulation of interleukin-10 production | 2.64E-09 |
| BP | positive regulation of CD4-positive, alpha-beta T cell activation | 2.64E-09 |
| BP | regulation of antigen receptor-mediated signaling pathway | 2.86E-09 |
| BP | production of molecular mediator of immune response | 3.02E-09 |
| BP | regulation of mononuclear cell migration | 3.75E-09 |
| BP | cytokine production involved in immune response | 3.81E-09 |
| BP | antigen processing and presentation of endogenous antigen | 3.87E-09 |
| BP | divalent metal ion transport | 4.42E-09 |
| BP | mast cell degranulation | 4.90E-09 |
| BP | regulation of CD4-positive, alpha-beta T cell differentiation | 4.90E-09 |
| BP | T cell apoptotic process | 4.90E-09 |
| BP | negative regulation of lymphocyte mediated immunity | 5.03E-09 |
| BP | positive regulation of CD4-positive, alpha-beta T cell differentiation | 5.43E-09 |
| BP | positive regulation of lymphocyte mediated immunity | 6.02E-09 |
| BP | divalent inorganic cation transport | 6.23E-09 |
| BP | T-helper cell differentiation | 6.30E-09 |
| BP | mast cell activation involved in immune response | 6.37E-09 |
| BP | positive regulation of T cell migration | 7.81E-09 |
| BP | T cell differentiation in thymus | 7.97E-09 |
| BP | macrophage activation | 9.39E-09 |
| BP | hemostasis | 1.18E-08 |
| BP | release of sequestered calcium ion into cytosol | 1.29E-08 |
| BP | negative regulation of leukocyte mediated cytotoxicity | 1.32E-08 |
| BP | tolerance induction | 1.48E-08 |
| BP | regulation of monocyte chemotaxis | 1.48E-08 |
| BP | calcium ion transmembrane import into cytosol | 1.58E-08 |
| BP | regulation of mast cell activation | 1.61E-08 |
| BP | dendritic cell differentiation | 1.61E-08 |
| BP | negative regulation of sequestering of calcium ion | 1.66E-08 |
| BP | defense response to virus | 1.85E-08 |
| BP | neuroinflammatory response | 2.03E-08 |
| BP | positive thymic T cell selection | 2.06E-08 |
| BP | regulation of sequestering of calcium ion | 2.14E-08 |
| BP | regulatory T cell differentiation | 2.14E-08 |
| BP | positive regulation of T cell mediated cytotoxicity | 2.20E-08 |
| BP | regulation of response to cytokine stimulus | 2.29E-08 |
| BP | negative regulation of interferon-gamma production | 2.92E-08 |
| BP | innate immune response-activating signal transduction | 3.00E-08 |
| BP | interleukin-6 production | 3.05E-08 |
| BP | sequestering of calcium ion | 3.09E-08 |
| BP | negative regulation of leukocyte differentiation | 3.10E-08 |
| BP | regulation of interleukin-4 production | 3.22E-08 |
| BP | regulation of small GTPase mediated signal transduction | 3.50E-08 |
| BP | myeloid cell homeostasis | 3.53E-08 |
| BP | positive regulation of tumor necrosis factor biosynthetic process | 3.62E-08 |
| BP | second-messenger-mediated signaling | 3.85E-08 |
| BP | regulation of neutrophil migration | 3.94E-08 |
| BP | immunological synapse formation | 4.02E-08 |
| BP | positive regulation of T cell apoptotic process | 4.02E-08 |
| BP | antigen processing and presentation of exogenous peptide antigen via MHC class I | 4.06E-08 |
| BP | regulation of natural killer cell mediated immunity | 4.54E-08 |
| BP | actin filament polymerization | 5.09E-08 |
| BP | STAT cascade | 5.13E-08 |
| BP | regulation of multi-organism process | 5.69E-08 |
| BP | positive regulation of lymphocyte chemotaxis | 5.71E-08 |
| BP | regulation of interleukin-6 production | 6.10E-08 |
| BP | lipopolysaccharide-mediated signaling pathway | 6.31E-08 |
| BP | positive regulation of neutrophil migration | 6.54E-08 |
| BP | platelet activation | 6.79E-08 |
| BP | microglial cell activation | 7.31E-08 |
| BP | leukocyte activation involved in inflammatory response | 7.31E-08 |
| BP | positive regulation of T cell mediated immunity | 7.31E-08 |
| BP | antigen processing and presentation of endogenous peptide antigen | 7.35E-08 |
| BP | negative regulation of natural killer cell mediated immunity | 7.35E-08 |
| BP | antigen processing and presentation of endogenous peptide antigen via MHC class I | 7.35E-08 |
| BP | negative regulation of defense response | 8.44E-08 |
| BP | negative regulation of cell killing | 8.75E-08 |
| BP | interleukin-2 biosynthetic process | 8.75E-08 |
| BP | regulation of defense response to virus by virus | 9.11E-08 |
| BP | negative regulation of B cell activation | 9.11E-08 |
| BP | JAK-STAT cascade | 9.29E-08 |
| BP | T cell activation via T cell receptor contact with antigen bound to MHC molecule on antigen presenting cell | 9.48E-08 |
| BP | dendritic cell apoptotic process | 9.48E-08 |
| BP | regulation of dendritic cell apoptotic process | 9.48E-08 |
| BP | regulation of peptide secretion | 1.07E-07 |
| BP | blood coagulation | 1.09E-07 |
| BP | positive regulation of B cell activation | 1.09E-07 |
| BP | regulation of mast cell degranulation | 1.25E-07 |
| BP | antigen processing and presentation via MHC class Ib | 1.27E-07 |
| BP | negative regulation of B cell proliferation | 1.27E-07 |
| BP | positive regulation of interleukin-4 production | 1.31E-07 |
| BP | lymphocyte homeostasis | 1.38E-07 |
| BP | regulation of cell morphogenesis | 1.40E-07 |
| BP | regulation of cytokine-mediated signaling pathway | 1.50E-07 |
| BP | Fc receptor mediated stimulatory signaling pathway | 1.51E-07 |
| BP | coagulation | 1.58E-07 |
| BP | regulation of natural killer cell activation | 1.70E-07 |
| BP | regulation of mast cell activation involved in immune response | 1.70E-07 |
| BP | regulation of regulatory T cell differentiation | 1.70E-07 |
| BP | positive regulation of antigen receptor-mediated signaling pathway | 1.91E-07 |
| BP | positive regulation of neutrophil chemotaxis | 1.91E-07 |
| BP | natural killer cell chemotaxis | 2.04E-07 |
| BP | negative thymic T cell selection | 2.04E-07 |
| BP | interleukin-1 beta secretion | 2.17E-07 |
| BP | regulation of protein secretion | 2.26E-07 |
| BP | interleukin-4 production | 2.28E-07 |
| BP | protein autophosphorylation | 2.50E-07 |
| BP | positive regulation of cytokine production involved in immune response | 2.66E-07 |
| BP | mature B cell differentiation | 2.75E-07 |
| BP | tumor necrosis factor-mediated signaling pathway | 2.75E-07 |
| BP | tumor necrosis factor biosynthetic process | 3.02E-07 |
| BP | regulation of tumor necrosis factor biosynthetic process | 3.02E-07 |
| BP | regulation of peptidyl-tyrosine phosphorylation | 3.05E-07 |
| BP | regulation of natural killer cell mediated cytotoxicity | 3.14E-07 |
| BP | regulation of granulocyte chemotaxis | 3.14E-07 |
| BP | regulation of T cell migration | 3.14E-07 |
| BP | negative regulation of interleukin-10 production | 3.38E-07 |
| BP | positive regulation of lymphocyte apoptotic process | 3.38E-07 |
| BP | positive regulation of monocyte chemotaxis | 3.38E-07 |
| BP | positive regulation of granulocyte chemotaxis | 3.87E-07 |
| BP | Fc receptor signaling pathway | 3.92E-07 |
| BP | dendritic cell antigen processing and presentation | 3.98E-07 |
| BP | interleukin-2-mediated signaling pathway | 3.98E-07 |
| BP | negative T cell selection | 3.98E-07 |
| BP | positive regulation of cytokine secretion | 4.14E-07 |
| BP | negative regulation of hemopoiesis | 4.16E-07 |
| BP | actin filament organization | 4.35E-07 |
| BP | positive regulation of production of molecular mediator of immune response | 4.41E-07 |
| BP | positive regulation of response to cytokine stimulus | 4.74E-07 |
| BP | regulation of cell adhesion mediated by integrin | 4.90E-07 |
| BP | regulation of interleukin-1 beta secretion | 4.90E-07 |
| BP | antigen processing and presentation of peptide antigen via MHC class I | 5.03E-07 |
| BP | regulation of interleukin-2 biosynthetic process | 5.22E-07 |
| BP | regulation of T-helper 1 type immune response | 5.36E-07 |
| BP | positive regulation of phagocytosis | 5.53E-07 |
| BP | Fc-gamma receptor signaling pathway | 5.63E-07 |
| BP | glial cell activation | 5.70E-07 |
| BP | peptidyl-tyrosine autophosphorylation | 6.64E-07 |
| BP | respiratory burst | 6.64E-07 |
| BP | negative regulation of leukocyte degranulation | 7.21E-07 |
| BP | positive regulation of interleukin-2 biosynthetic process | 7.21E-07 |
| BP | cellular response to interleukin-2 | 7.21E-07 |
| BP | production of molecular mediator involved in inflammatory response | 7.58E-07 |
| BP | negative regulation of tumor necrosis factor superfamily cytokine production | 7.58E-07 |
| BP | positive regulation of natural killer cell activation | 7.83E-07 |
| BP | regulation of myeloid cell differentiation | 8.03E-07 |
| BP | regulation of actin polymerization or depolymerization | 8.08E-07 |
| BP | negative regulation of alpha-beta T cell activation | 8.48E-07 |
| BP | maintenance of location | 8.56E-07 |
| BP | regulation of actin filament length | 8.80E-07 |
| BP | regulation of metal ion transport | 9.67E-07 |
| BP | interleukin-1 secretion | 9.70E-07 |
| BP | regulation of myeloid cell apoptotic process | 9.86E-07 |
| BP | lysosome localization | 1.03E-06 |
| BP | regulation of calcium ion transport into cytosol | 1.07E-06 |
| BP | eosinophil chemotaxis | 1.15E-06 |
| BP | antigen processing and presentation of exogenous peptide antigen via MHC class I, TAP-dependent | 1.19E-06 |
| BP | integrin-mediated signaling pathway | 1.21E-06 |
| BP | negative regulation of natural killer cell mediated cytotoxicity | 1.23E-06 |
| BP | response to interleukin-2 | 1.23E-06 |
| BP | regulation of cysteine-type endopeptidase activity | 1.29E-06 |
| BP | B cell homeostasis | 1.31E-06 |
| BP | regulation of neutrophil chemotaxis | 1.31E-06 |
| BP | positive regulation of STAT cascade | 1.85E-06 |
| BP | regulation of interleukin-1 secretion | 1.96E-06 |
| BP | immune response-regulating cell surface receptor signaling pathway involved in phagocytosis | 2.05E-06 |
| BP | Fc-gamma receptor signaling pathway involved in phagocytosis | 2.05E-06 |
| BP | positive regulation of protein secretion | 2.48E-06 |
| BP | positive regulation of peptide secretion | 2.53E-06 |
| BP | type I interferon signaling pathway | 2.68E-06 |
| BP | cellular response to type I interferon | 2.68E-06 |
| BP | myeloid cell apoptotic process | 2.89E-06 |
| BP | cell adhesion mediated by integrin | 2.97E-06 |
| BP | positive regulation of interleukin-6 production | 3.02E-06 |
| BP | neutrophil homeostasis | 3.13E-06 |
| BP | regulation of tolerance induction | 3.13E-06 |
| BP | interleukin-8 production | 3.16E-06 |
| BP | positive regulation of mononuclear cell migration | 3.17E-06 |
| BP | leukocyte adhesion to vascular endothelial cell | 3.17E-06 |
| BP | regulation of actin filament polymerization | 3.57E-06 |
| BP | positive regulation of secretion by cell | 3.69E-06 |
| BP | regulation of calcium-mediated signaling | 3.82E-06 |
| BP | negative regulation of adaptive immune response | 3.87E-06 |
| BP | negative regulation of T cell differentiation | 3.87E-06 |
| BP | interleukin-12 secretion | 3.98E-06 |
| BP | negative regulation of tumor necrosis factor production | 3.99E-06 |
| BP | response to type I interferon | 4.29E-06 |
| BP | leukocyte tethering or rolling | 4.29E-06 |
| BP | type 2 immune response | 4.66E-06 |
| BP | regulation of T cell chemotaxis | 4.72E-06 |
| BP | regulation of endocytosis | 5.50E-06 |
| BP | regulation of myeloid leukocyte differentiation | 5.62E-06 |
| BP | positive regulation of phosphatidylinositol 3-kinase signaling | 5.96E-06 |
| BP | negative regulation of T cell apoptotic process | 6.91E-06 |
| BP | regulation of interleukin-8 production | 6.96E-06 |
| BP | negative regulation of cytokine secretion | 6.96E-06 |
| BP | response to interleukin-1 | 7.07E-06 |
| BP | regulation of dendritic cell antigen processing and presentation | 7.12E-06 |
| BP | negative regulation of myeloid leukocyte mediated immunity | 7.12E-06 |
| BP | regulation of neutrophil activation | 7.12E-06 |
| BP | positive regulation of endocytosis | 7.20E-06 |
| BP | positive regulation of JAK-STAT cascade | 7.58E-06 |
| BP | regulation of viral process | 7.58E-06 |
| BP | I-kappaB kinase/NF-kappaB signaling | 8.89E-06 |
| BP | macrophage chemotaxis | 8.98E-06 |
| BP | positive regulation of peptidyl-tyrosine phosphorylation | 9.16E-06 |
| BP | positive regulation of cytokine-mediated signaling pathway | 9.64E-06 |
| BP | cell-substrate adhesion | 9.75E-06 |
| BP | myeloid dendritic cell activation | 9.82E-06 |
| BP | mature B cell differentiation involved in immune response | 9.86E-06 |
| BP | positive regulation of calcium ion transport | 1.01E-05 |
| BP | regulation of T cell receptor signaling pathway | 1.10E-05 |
| BP | regulation of phosphatidylinositol 3-kinase signaling | 1.11E-05 |
| BP | calcium ion transmembrane transport | 1.17E-05 |
| BP | positive regulation of B cell differentiation | 1.19E-05 |
| BP | negative regulation of lymphocyte apoptotic process | 1.26E-05 |
| BP | negative regulation of CD4-positive, alpha-beta T cell activation | 1.26E-05 |
| BP | regulation of regulated secretory pathway | 1.27E-05 |
| BP | pattern recognition receptor signaling pathway | 1.31E-05 |
| BP | regulation of type I interferon production | 1.34E-05 |
| BP | negative regulation of adaptive immune response based on somatic recombination of immune receptors built from immunoglobulin superfamily domains | 1.34E-05 |
| BP | positive regulation of T-helper 1 type immune response | 1.37E-05 |
| BP | positive regulation of cell adhesion mediated by integrin | 1.37E-05 |
| BP | CD4-positive or CD8-positive, alpha-beta T cell lineage commitment | 1.37E-05 |
| BP | nitric-oxide synthase biosynthetic process | 1.37E-05 |
| BP | regulation of nitric-oxide synthase biosynthetic process | 1.37E-05 |
| BP | positive regulation of secretion | 1.42E-05 |
| BP | regulation of I-kappaB kinase/NF-kappaB signaling | 1.43E-05 |
| BP | phosphatidylinositol-mediated signaling | 1.58E-05 |
| BP | macrophage migration | 1.58E-05 |
| BP | type I interferon production | 1.60E-05 |
| BP | regulation of type 2 immune response | 1.61E-05 |
| BP | regulation of actin cytoskeleton organization | 1.64E-05 |
| BP | regulation of STAT cascade | 1.71E-05 |
| BP | positive regulation of I-kappaB kinase/NF-kappaB signaling | 1.82E-05 |
| BP | osteoclast differentiation | 1.85E-05 |
| BP | natural killer cell differentiation | 1.88E-05 |
| BP | complement receptor mediated signaling pathway | 1.89E-05 |
| BP | negative regulation of mast cell activation | 1.89E-05 |
| BP | G protein-coupled purinergic nucleotide receptor signaling pathway | 1.89E-05 |
| BP | interleukin-15-mediated signaling pathway | 1.89E-05 |
| BP | cellular response to interleukin-15 | 1.89E-05 |
| BP | regulation of symbiosis, encompassing mutualism through parasitism | 1.92E-05 |
| BP | inositol lipid-mediated signaling | 1.96E-05 |
| BP | regulation of T cell cytokine production | 2.03E-05 |
| BP | positive regulation of myeloid leukocyte mediated immunity | 2.03E-05 |
| BP | regulation of cellular component size | 2.11E-05 |
| BP | negative regulation of lymphocyte differentiation | 2.16E-05 |
| BP | positive regulation of actin filament polymerization | 2.28E-05 |
| BP | negative regulation of viral process | 2.28E-05 |
| BP | inflammatory cell apoptotic process | 2.52E-05 |
| BP | positive regulation of T-helper cell differentiation | 2.52E-05 |
| BP | myeloid cell development | 2.60E-05 |
| BP | macrophage differentiation | 2.80E-05 |
| BP | gamma-delta T cell activation | 2.88E-05 |
| BP | response to interleukin-15 | 2.88E-05 |
| BP | negative regulation of multi-organism process | 2.92E-05 |
| BP | membrane invagination | 2.93E-05 |
| BP | phagocytosis, engulfment | 2.96E-05 |
| BP | positive regulation of interleukin-1 beta secretion | 3.14E-05 |
| BP | negative regulation of secretion by cell | 3.30E-05 |
| BP | negative regulation of interleukin-2 production | 3.33E-05 |
| BP | positive regulation of chemokine production | 3.35E-05 |
| BP | regulation of JAK-STAT cascade | 3.45E-05 |
| BP | regulation of MAP kinase activity | 3.51E-05 |
| BP | cell-cell recognition | 3.77E-05 |
| BP | negative regulation of exocytosis | 3.85E-05 |
| BP | chemokine production | 4.15E-05 |
| BP | positive regulation of protein kinase B signaling | 4.16E-05 |
| BP | positive regulation of interleukin-6 biosynthetic process | 4.21E-05 |
| BP | positive regulation of nitric-oxide synthase biosynthetic process | 4.21E-05 |
| BP | erythrocyte homeostasis | 4.22E-05 |
| BP | defense response to protozoan | 4.34E-05 |
| BP | positive regulation of leukocyte degranulation | 4.34E-05 |
| BP | regulation of DNA-binding transcription factor activity | 4.39E-05 |
| BP | positive regulation of establishment of protein localization | 4.53E-05 |
| BP | regulation of T-helper cell differentiation | 4.70E-05 |
| BP | regulation of actin filament-based process | 4.72E-05 |
| BP | regulation of exocytosis | 4.77E-05 |
| BP | negative regulation of secretion | 5.00E-05 |
| BP | tissue remodeling | 5.11E-05 |
| BP | response to protozoan | 5.57E-05 |
| BP | negative regulation of regulated secretory pathway | 5.57E-05 |
| BP | regulation of interleukin-8 secretion | 5.57E-05 |
| BP | regulation of actin filament organization | 5.69E-05 |
| BP | protein polymerization | 5.96E-05 |
| BP | positive regulation of type 2 immune response | 5.99E-05 |
| BP | positive regulation of T cell chemotaxis | 5.99E-05 |
| BP | negative regulation of myeloid cell apoptotic process | 5.99E-05 |
| BP | positive regulation of interleukin-8 production | 6.30E-05 |
| BP | positive regulation of protein transport | 6.35E-05 |
| BP | plasma membrane invagination | 6.42E-05 |
| BP | viral life cycle | 6.45E-05 |
| BP | regulation of actin cytoskeleton reorganization | 6.85E-05 |
| BP | T cell lineage commitment | 7.07E-05 |
| BP | regulation of macrophage chemotaxis | 7.07E-05 |
| BP | defense response to bacterium | 7.08E-05 |
| BP | phosphatidylinositol 3-kinase signaling | 8.09E-05 |
| BP | positive regulation of interleukin-1 secretion | 8.20E-05 |
| BP | positive regulation of protein complex assembly | 8.21E-05 |
| BP | Ras protein signal transduction | 8.25E-05 |
| BP | gliogenesis | 8.45E-05 |
| BP | positive regulation of NF-kappaB transcription factor activity | 8.70E-05 |
| BP | regulation of viral life cycle | 8.70E-05 |
| BP | regulation of macrophage migration | 9.75E-05 |
| BP | regulation of chemokine production | 0.000103542 |
| BP | regulation of release of sequestered calcium ion into cytosol | 0.000103542 |
| BP | regulation of interleukin-6 biosynthetic process | 0.000110229 |
| BP | T cell cytokine production | 0.000115291 |
| BP | establishment of T cell polarity | 0.000115964 |
| BP | natural killer cell proliferation | 0.000115964 |
| BP | multi-organism membrane organization | 0.000115964 |
| BP | negative regulation of alpha-beta T cell proliferation | 0.000115964 |
| BP | response to interleukin-9 | 0.000115964 |
| BP | immunoglobulin production | 0.000124889 |
| BP | regulation of neuroinflammatory response | 0.000135675 |
| BP | interleukin-6 biosynthetic process | 0.000135689 |
| BP | regulation of B cell receptor signaling pathway | 0.000135689 |
| BP | regulation of syncytium formation by plasma membrane fusion | 0.000135689 |
| BP | interleukin-8 secretion | 0.000135689 |
| BP | regulation of cysteine-type endopeptidase activity involved in apoptotic process | 0.000138272 |
| BP | positive regulation of MAP kinase activity | 0.000146103 |
| BP | regulation of macrophage activation | 0.00014671 |
| BP | alpha-beta T cell lineage commitment | 0.000148558 |
| BP | chronic inflammatory response | 0.000148558 |
| BP | negative regulation of T cell mediated immunity | 0.000148558 |
| BP | positive regulation of superoxide anion generation | 0.000148558 |
| BP | T-helper 1 cell differentiation | 0.000148558 |
| BP | regulation of protein polymerization | 0.000163413 |
| BP | positive regulation of natural killer cell mediated cytotoxicity | 0.000165591 |
| BP | regulation of acute inflammatory response | 0.000174076 |
| BP | establishment of lymphocyte polarity | 0.000178159 |
| BP | regulation of cell-cell adhesion mediated by integrin | 0.000178159 |
| BP | CD4-positive, alpha-beta T cell proliferation | 0.000178159 |
| BP | interleukin-35-mediated signaling pathway | 0.000178159 |
| BP | interleukin-10 secretion | 0.000178159 |
| BP | regulation of CD4-positive, alpha-beta T cell proliferation | 0.000178159 |
| BP | cellular response to interleukin-1 | 0.000179028 |
| BP | acute inflammatory response | 0.000182314 |
| BP | cortical actin cytoskeleton organization | 0.000185205 |
| BP | positive regulation of calcium-mediated signaling | 0.000185205 |
| BP | B cell mediated immunity | 0.00019246 |
| BP | interleukin-8 biosynthetic process | 0.000193495 |
| BP | positive regulation of cytokine production involved in inflammatory response | 0.000193495 |
| BP | positive regulation of macrophage migration | 0.000193495 |
| BP | regulation of endopeptidase activity | 0.000205928 |
| BP | B cell activation involved in immune response | 0.000210033 |
| BP | superoxide metabolic process | 0.000210033 |
| BP | positive regulation of protein serine/threonine kinase activity | 0.000226402 |
| BP | modification of morphology or physiology of other organism | 0.000240602 |
| BP | cellular response to interleukin-4 | 0.000240889 |
| BP | positive regulation of myeloid cell differentiation | 0.000248024 |
| BP | acute inflammatory response to antigenic stimulus | 0.000248091 |
| BP | purinergic nucleotide receptor signaling pathway | 0.000248091 |
| BP | regulation of myoblast fusion | 0.000248091 |
| BP | positive regulation of inflammatory response to antigenic stimulus | 0.000261275 |
| BP | response to macrophage colony-stimulating factor | 0.000261275 |
| BP | cellular response to macrophage colony-stimulating factor stimulus | 0.000261275 |
| BP | positive regulation of interleukin-8 biosynthetic process | 0.000261275 |
| BP | NK T cell activation | 0.000261275 |
| BP | leukocyte aggregation | 0.000261275 |
| BP | toll-like receptor signaling pathway | 0.000263504 |
| BP | negative regulation of cysteine-type endopeptidase activity | 0.000271365 |
| BP | negative regulation of type I interferon production | 0.000285936 |
| BP | positive regulation of natural killer cell mediated immunity | 0.000287453 |
| BP | regulation of superoxide anion generation | 0.000313641 |
| BP | integrin activation | 0.000313641 |
| BP | negative regulation of phagocytosis | 0.000313641 |
| BP | regulation of erythrocyte differentiation | 0.000327891 |
| BP | response to interleukin-4 | 0.000340795 |
| BP | bone resorption | 0.000342735 |
| BP | regulation of calcium ion transmembrane transport | 0.000366092 |
| BP | regulation of acute inflammatory response to antigenic stimulus | 0.000368985 |
| BP | regulation of natural killer cell differentiation | 0.000368985 |
| BP | negative regulation of myeloid leukocyte differentiation | 0.000374641 |
| BP | cortical cytoskeleton organization | 0.000374641 |
| BP | regulation of protein complex assembly | 0.000383785 |
| BP | erythrocyte differentiation | 0.00038833 |
| BP | response to prostaglandin E | 0.000391512 |
| BP | bone cell development | 0.000401582 |
| BP | cell recognition | 0.000425736 |
| BP | cytokine production involved in inflammatory response | 0.000426575 |
| BP | regulation of humoral immune response | 0.000426744 |
| BP | positive regulation of protein polymerization | 0.000426744 |
| BP | regulation of lipid kinase activity | 0.000427085 |
| BP | regulation of osteoclast differentiation | 0.000427085 |
| BP | membrane fusion | 0.000442624 |
| BP | innate immune response activating cell surface receptor signaling pathway | 0.000451016 |
| BP | MyD88-dependent toll-like receptor signaling pathway | 0.000470513 |
| BP | monocyte differentiation | 0.000470513 |
| BP | superoxide anion generation | 0.000470513 |
| BP | positive regulation of lipid kinase activity | 0.000470513 |
| BP | mononuclear cell differentiation | 0.000470513 |
| BP | G protein-coupled purinergic receptor signaling pathway | 0.000483132 |
| BP | regulation of NIK/NF-kappaB signaling | 0.000485405 |
| BP | immunoglobulin mediated immune response | 0.000495856 |
| BP | CD8-positive, alpha-beta T cell differentiation | 0.000505075 |
| BP | T-helper 2 cell differentiation | 0.000505075 |
| BP | regulation of MHC class II biosynthetic process | 0.000505075 |
| BP | positive regulation of regulatory T cell differentiation | 0.000505075 |
| BP | positive regulation of T cell receptor signaling pathway | 0.000505075 |
| BP | JAK-STAT cascade involved in growth hormone signaling pathway | 0.000505075 |
| BP | detection of other organism | 0.000505075 |
| BP | regulation of peptidase activity | 0.000528156 |
| BP | T cell homeostasis | 0.000548321 |
| BP | positive regulation of endopeptidase activity | 0.000554089 |
| BP | actin cytoskeleton reorganization | 0.00057684 |
| BP | negative regulation of cytokine production involved in immune response | 0.000589993 |
| BP | regulation of response to biotic stimulus | 0.000594453 |
| BP | regulation of neuron death | 0.000631851 |
| BP | positive regulation of cell-substrate adhesion | 0.000645696 |
| BP | viral entry into host cell | 0.000645696 |
| BP | negative regulation of viral life cycle | 0.000660672 |
| BP | germinal center formation | 0.000673416 |
| BP | positive regulation of macrophage chemotaxis | 0.000673416 |
| BP | regulation of granulocyte differentiation | 0.000673416 |
| BP | activation of Janus kinase activity | 0.000673416 |
| BP | MHC class II biosynthetic process | 0.000673416 |
| BP | positive regulation of interleukin-8 secretion | 0.000673416 |
| BP | protein kinase B signaling | 0.000683122 |
| BP | stress-activated protein kinase signaling cascade | 0.000684111 |
| BP | Rho protein signal transduction | 0.000685984 |
| BP | viral genome replication | 0.000692007 |
| BP | negative regulation of interleukin-6 production | 0.000694547 |
| BP | positive regulation of regulated secretory pathway | 0.000694547 |
| BP | positive regulation of defense response to virus by host | 0.000713639 |
| BP | inositol trisphosphate metabolic process | 0.000713639 |
| BP | tyrosine phosphorylation of STAT protein | 0.000719782 |
| BP | muscle cell migration | 0.000726904 |
| BP | NIK/NF-kappaB signaling | 0.000729187 |
| BP | tissue homeostasis | 0.000768689 |
| BP | regulation of phospholipase activity | 0.000785834 |
| BP | cytolysis | 0.000842757 |
| BP | negative regulation of cytokine biosynthetic process | 0.000842757 |
| BP | regulation of bone resorption | 0.000842757 |
| BP | regulation of tissue remodeling | 0.000850952 |
| BP | negative regulation of osteoclast differentiation | 0.000855668 |
| BP | positive regulation of syncytium formation by plasma membrane fusion | 0.000855668 |
| BP | positive regulation of tyrosine phosphorylation of STAT protein | 0.000864115 |
| BP | inositol phosphate metabolic process | 0.000864115 |
| BP | regulation of phosphatidylinositol 3-kinase activity | 0.000871038 |
| BP | T-helper cell lineage commitment | 0.000877931 |
| BP | regulation of inositol phosphate biosynthetic process | 0.000877931 |
| BP | negative regulation of interleukin-12 production | 0.000877931 |
| BP | cell-cell adhesion mediated by integrin | 0.000877931 |
| BP | regulation of membrane invagination | 0.000877931 |
| BP | positive regulation of ion transport | 0.00088142 |
| BP | myoblast fusion | 0.000963971 |
| BP | activated T cell proliferation | 0.000963971 |
| BP | syncytium formation by plasma membrane fusion | 0.000971618 |
| BP | cell-cell fusion | 0.000971618 |
| BP | neuron death | 0.000973577 |
| BP | bone remodeling | 0.001000922 |
| BP | response to alcohol | 0.001014338 |
| BP | regulation of cellular extravasation | 0.00101772 |
| BP | response to interferon-beta | 0.00101772 |
| BP | negative regulation of antigen receptor-mediated signaling pathway | 0.00101772 |
| BP | negative regulation of peptide secretion | 0.001035736 |
| BP | negative regulation of myeloid cell differentiation | 0.001083557 |
| BP | positive regulation of immunoglobulin production | 0.001098152 |
| BP | inositol phosphate biosynthetic process | 0.001098152 |
| BP | positive regulation of cysteine-type endopeptidase activity | 0.001098289 |
| BP | positive regulation of myelination | 0.001122572 |
| BP | megakaryocyte development | 0.001122572 |
| BP | CD4-positive, alpha-beta T cell lineage commitment | 0.001122572 |
| BP | regulation of respiratory burst | 0.001122572 |
| BP | positive regulation of leukocyte adhesion to vascular endothelial cell | 0.001122572 |
| BP | regulation of cysteine-type endopeptidase activity involved in apoptotic signaling pathway | 0.001122572 |
| BP | regulation of supramolecular fiber organization | 0.001123456 |
| BP | JNK cascade | 0.001173979 |
| BP | syncytium formation | 0.001200066 |
| BP | positive regulation of dephosphorylation | 0.001200066 |
| BP | regulation of cell-substrate adhesion | 0.001230097 |
| BP | positive regulation of phospholipase C activity | 0.001246198 |
| BP | positive regulation of nitric oxide biosynthetic process | 0.001246198 |
| BP | reactive oxygen species metabolic process | 0.001270301 |
| BP | positive regulation of DNA-binding transcription factor activity | 0.001278856 |
| BP | activation of MAPK activity | 0.001305047 |
| BP | positive regulation of phospholipase activity | 0.001329029 |
| BP | negative regulation of viral genome replication | 0.001329029 |
| BP | activation of protein kinase activity | 0.001348023 |
| BP | vesicle fusion | 0.001365108 |
| BP | stimulatory C-type lectin receptor signaling pathway | 0.001380511 |
| BP | homeostasis of number of cells within a tissue | 0.001408653 |
| BP | positive regulation of nitric oxide metabolic process | 0.001409028 |
| BP | interferon-gamma biosynthetic process | 0.00141129 |
| BP | monocyte chemotactic protein-1 production | 0.00141129 |
| BP | regulation of monocyte chemotactic protein-1 production | 0.00141129 |
| BP | positive regulation of NIK/NF-kappaB signaling | 0.001474956 |
| BP | positive regulation of peptidase activity | 0.001485365 |
| BP | entry into host cell | 0.001498327 |
| BP | entry into host | 0.001498327 |
| BP | entry into cell of other organism involved in symbiotic interaction | 0.001498327 |
| BP | entry into other organism involved in symbiotic interaction | 0.001498327 |
| BP | vascular endothelial growth factor receptor signaling pathway | 0.001583521 |
| BP | regulation of phospholipase C activity | 0.001587586 |
| BP | regulation of tumor necrosis factor-mediated signaling pathway | 0.001619282 |
| BP | heterotypic cell-cell adhesion | 0.001619282 |
| BP | regulation of protein kinase B signaling | 0.001638703 |
| BP | positive regulation of acute inflammatory response | 0.001640996 |
| BP | activation of phospholipase C activity | 0.001640996 |
| BP | regulation of platelet activation | 0.001640996 |
| BP | response to prostaglandin | 0.001640996 |
| BP | positive regulation of cold-induced thermogenesis | 0.001702778 |
| BP | regulation of blood coagulation | 0.001741214 |
| BP | positive regulation of T cell cytokine production | 0.001748019 |
| BP | regulation of interleukin-8 biosynthetic process | 0.001748019 |
| BP | positive regulation of myeloid leukocyte cytokine production involved in immune response | 0.001748019 |
| BP | detection of external biotic stimulus | 0.001748019 |
| BP | positive regulation of myoblast fusion | 0.001748019 |
| BP | apoptotic cell clearance | 0.001782834 |
| BP | regulation of cation transmembrane transport | 0.001848479 |
| BP | regulation of hemostasis | 0.001887861 |
| BP | purinergic receptor signaling pathway | 0.001900275 |
| BP | positive regulation of phosphatidylinositol 3-kinase activity | 0.001900275 |
| BP | regulation of immunoglobulin production | 0.001956593 |
| BP | cellular response to drug | 0.002001816 |
| BP | phospholipase C-activating G protein-coupled receptor signaling pathway | 0.002104276 |
| BP | organelle membrane fusion | 0.002104276 |
| BP | positive regulation of JNK cascade | 0.00212536 |
| BP | cytokine secretion involved in immune response | 0.00213665 |
| BP | negative regulation of tissue remodeling | 0.00213665 |
| BP | cellular response to interferon-beta | 0.00213665 |
| BP | regulation of microglial cell activation | 0.00213665 |
| BP | negative regulation of chemotaxis | 0.002144427 |
| BP | granulocyte differentiation | 0.002188279 |
| BP | regulation of bone remodeling | 0.002227339 |
| BP | positive regulation of phospholipid metabolic process | 0.002227339 |
| BP | negative regulation of protein secretion | 0.00224804 |
| BP | defense response to Gram-positive bacterium | 0.002253699 |
| BP | serotonin secretion | 0.002326556 |
| BP | interleukin-18 production | 0.002326556 |
| BP | positive regulation of MHC class II biosynthetic process | 0.002326556 |
| BP | regulation of T-helper 1 cell differentiation | 0.002326556 |
| BP | positive regulation of B cell receptor signaling pathway | 0.002326556 |
| BP | regulation of NK T cell activation | 0.002326556 |
| BP | regulation of macrophage apoptotic process | 0.002326556 |
| BP | regulation of JNK cascade | 0.00238658 |
| BP | regulation of tyrosine phosphorylation of STAT protein | 0.002386917 |
| BP | cellular response to calcium ion | 0.002386917 |
| BP | positive regulation of reactive oxygen species metabolic process | 0.00241144 |
| BP | negative regulation of neuron death | 0.002463261 |
| BP | positive regulation of phosphatase activity | 0.002506811 |
| BP | regulation of interleukin-17 production | 0.002506811 |
| BP | interaction with host | 0.002573726 |
| BP | regulation of smooth muscle cell migration | 0.002574426 |
| BP | negative regulation of cysteine-type endopeptidase activity involved in apoptotic process | 0.002574426 |
| BP | regulation of coagulation | 0.002574426 |
| BP | negative regulation of bone mineralization | 0.002581016 |
| BP | negative regulation of production of molecular mediator of immune response | 0.002857683 |
| BP | multicellular organismal homeostasis | 0.003014474 |
| BP | regulation of B cell mediated immunity | 0.003044314 |
| BP | regulation of immunoglobulin mediated immune response | 0.003044314 |
| BP | interferon-gamma secretion | 0.003084878 |
| BP | negative regulation of neuron apoptotic process | 0.003110199 |
| BP | T cell tolerance induction | 0.003132205 |
| BP | facial nerve development | 0.003132205 |
| BP | cranial nerve structural organization | 0.003132205 |
| BP | facial nerve morphogenesis | 0.003132205 |
| BP | regulation of T-helper 2 cell differentiation | 0.003132205 |
| BP | negative regulation of activated T cell proliferation | 0.003132205 |
| BP | interleukin-27-mediated signaling pathway | 0.003132205 |
| BP | synapse pruning | 0.003132205 |
| BP | macrophage derived foam cell differentiation | 0.00324271 |
| BP | foam cell differentiation | 0.00324271 |
| BP | regulation of superoxide metabolic process | 0.00324271 |
| BP | regulation of wound healing | 0.003276762 |
| BP | regulation of Ras protein signal transduction | 0.00329563 |
| BP | response to fungus | 0.003360837 |
| BP | ruffle organization | 0.003360837 |
| BP | regulation of protein tyrosine kinase activity | 0.003442307 |
| BP | regulation of phospholipid metabolic process | 0.003442307 |
| BP | regulation of toll-like receptor signaling pathway | 0.003580528 |
| BP | detection of biotic stimulus | 0.003651902 |
| BP | inositol trisphosphate biosynthetic process | 0.003651902 |
| BP | ribonucleoside catabolic process | 0.003651902 |
| BP | positive regulation of B cell mediated immunity | 0.003663708 |
| BP | positive regulation of immunoglobulin mediated immune response | 0.003663708 |
| BP | regulation of activated T cell proliferation | 0.003663708 |
| BP | regulation of defense response to virus by host | 0.003663708 |
| BP | negative regulation of peptidyl-tyrosine phosphorylation | 0.003701215 |
| BP | positive regulation of stress-activated protein kinase signaling cascade | 0.003835688 |
| BP | negative regulation of response to external stimulus | 0.003885617 |
| BP | positive regulation of myeloid leukocyte differentiation | 0.00406651 |
| BP | positive regulation of calcium ion transport into cytosol | 0.00406651 |
| BP | response to amyloid-beta | 0.00406651 |
| BP | cytidine catabolic process | 0.004089181 |
| BP | cytidine deamination | 0.004089181 |
| BP | cytidine to uridine editing | 0.004089181 |
| BP | negative regulation by host of viral transcription | 0.004089181 |
| BP | cytidine metabolic process | 0.004089181 |
| BP | regulation of Fc receptor mediated stimulatory signaling pathway | 0.004089181 |
| BP | regulation of thymocyte apoptotic process | 0.004089181 |
| BP | positive regulation of monocyte chemotactic protein-1 production | 0.004089181 |
| BP | macrophage apoptotic process | 0.004089181 |
| BP | T-helper 17 cell lineage commitment | 0.004089181 |
| BP | regulation of dendritic cell differentiation | 0.004089181 |
| BP | positive regulation of myotube differentiation | 0.004122488 |
| BP | interleukin-17 production | 0.004122488 |
| BP | positive regulation of lipase activity | 0.004193078 |
| BP | regulation of plasma membrane bounded cell projection assembly | 0.004205886 |
| BP | smooth muscle cell migration | 0.00423016 |
| BP | regulation of inflammatory response to antigenic stimulus | 0.004285656 |
| BP | growth hormone receptor signaling pathway | 0.004285656 |
| BP | myotube differentiation | 0.004519954 |
| BP | antimicrobial humoral immune response mediated by antimicrobial peptide | 0.004527557 |
| BP | regulation of cell projection assembly | 0.004604216 |
| BP | immunoglobulin production involved in immunoglobulin mediated immune response | 0.004876133 |
| BP | positive regulation of gliogenesis | 0.004881779 |
| BP | positive regulation of ion transmembrane transport | 0.004882527 |
| BP | cell-matrix adhesion | 0.004970766 |
| BP | interleukin-13 production | 0.004989592 |
| BP | cellular response to growth hormone stimulus | 0.004989592 |
| BP | regulation of response to wounding | 0.005032163 |
| BP | positive regulation of apoptotic signaling pathway | 0.005032163 |
| BP | protein homotetramerization | 0.005150023 |
| BP | positive regulation of release of sequestered calcium ion into cytosol | 0.005160575 |
| BP | response to interleukin-7 | 0.005160575 |
| BP | cellular response to interleukin-7 | 0.005160575 |
| BP | positive regulation of cytoskeleton organization | 0.005166202 |
| BP | macrophage activation involved in immune response | 0.00520524 |
| BP | respiratory burst involved in defense response | 0.00520524 |
| BP | regulation of integrin activation | 0.00520524 |
| BP | DNA deamination | 0.00520524 |
| BP | negative regulation of bone resorption | 0.00520524 |
| BP | neutrophil extravasation | 0.00520524 |
| BP | positive regulation of reactive oxygen species biosynthetic process | 0.005322606 |
| BP | positive regulation of supramolecular fiber organization | 0.005478537 |
| BP | protein homooligomerization | 0.005479143 |
| BP | regulation of viral genome replication | 0.005488318 |
| BP | regulation of ion transmembrane transport | 0.005674481 |
| BP | positive regulation of protein autophosphorylation | 0.005767038 |
| BP | T-helper 17 cell differentiation | 0.005767038 |
| BP | protein processing | 0.0058919 |
| BP | positive regulation of type I interferon production | 0.006070039 |
| BP | platelet aggregation | 0.006304237 |
| BP | regulation of glycoprotein biosynthetic process | 0.006371178 |
| BP | regulation of myelination | 0.006371178 |
| BP | leukocyte migration involved in inflammatory response | 0.006487103 |
| BP | regulation of macrophage cytokine production | 0.006487103 |
| BP | negative regulation of single stranded viral RNA replication via double stranded DNA intermediate | 0.006487103 |
| BP | pyrimidine ribonucleoside catabolic process | 0.006487103 |
| BP | positive regulation of killing of cells of other organism | 0.006487103 |
| BP | regulation of Rho protein signal transduction | 0.006513886 |
| BP | regulation of lipase activity | 0.00660538 |
| BP | positive regulation of macrophage activation | 0.006621189 |
| BP | regulation of neuron apoptotic process | 0.006941904 |
| BP | cellular response to amyloid-beta | 0.007045518 |
| BP | ERBB signaling pathway | 0.007177638 |
| BP | positive regulation of multi-organism process | 0.007365903 |
| BP | positive regulation of wound healing | 0.007411234 |
| BP | exocytic process | 0.007461766 |
| BP | cold-induced thermogenesis | 0.007528541 |
| BP | regulation of cold-induced thermogenesis | 0.007528541 |
| BP | regulation of interferon-alpha production | 0.007555101 |
| BP | regulation of leukocyte adhesion to vascular endothelial cell | 0.007555101 |
| BP | stress-activated MAPK cascade | 0.007653217 |
| BP | positive regulation of protein dephosphorylation | 0.007768145 |
| BP | epithelial cell differentiation involved in kidney development | 0.007768145 |
| BP | antimicrobial humoral response | 0.00784628 |
| BP | positive regulation of smooth muscle cell proliferation | 0.007886572 |
| BP | positive regulation of cation transmembrane transport | 0.007892542 |
| BP | regulation of granulocyte macrophage colony-stimulating factor production | 0.007940525 |
| BP | positive regulation of mast cell activation involved in immune response | 0.007940525 |
| BP | positive regulation of mast cell degranulation | 0.007940525 |
| BP | negative regulation of bone remodeling | 0.007940525 |
| BP | regulation of phagocytosis, engulfment | 0.007940525 |
| BP | homotypic cell-cell adhesion | 0.00797425 |
| BP | cellular response to metal ion | 0.007984701 |
| BP | regulation of myotube differentiation | 0.008014392 |
| BP | neuron apoptotic process | 0.008317693 |
| BP | regulation of stress-activated protein kinase signaling cascade | 0.008317693 |
| BP | negative regulation of ossification | 0.008512221 |
| BP | nitric oxide metabolic process | 0.008512221 |
| BP | intrinsic apoptotic signaling pathway in response to DNA damage by p53 class mediator | 0.008540714 |
| BP | substrate-dependent cell migration | 0.00857168 |
| BP | interleukin-6-mediated signaling pathway | 0.00857168 |
| BP | T-helper 17 type immune response | 0.00857168 |
| BP | carbohydrate derivative catabolic process | 0.008643497 |
| BP | positive regulation of neurological system process | 0.008652055 |
| BP | organelle fusion | 0.008691217 |
| BP | negative regulation of protein transport | 0.00898842 |
| BP | positive regulation of Ras protein signal transduction | 0.009325258 |
| BP | intrinsic apoptotic signaling pathway in response to DNA damage | 0.009345972 |
| BP | glial cell differentiation | 0.009357149 |
| BP | inflammatory response to antigenic stimulus | 0.009364846 |
| BP | heterophilic cell-cell adhesion via plasma membrane cell adhesion molecules | 0.009364846 |
| BP | cellular response to interleukin-6 | 0.009364846 |
| BP | regulation of cytokine production involved in inflammatory response | 0.009364846 |
| BP | regulation of cytokine secretion involved in immune response | 0.009570355 |
| BP | serotonin transport | 0.009570355 |
| BP | complement activation, alternative pathway | 0.009570355 |
| BP | macrophage cytokine production | 0.009570355 |
| BP | granulocyte macrophage colony-stimulating factor production | 0.009570355 |
| BP | negative regulation of macrophage activation | 0.009570355 |
| BP | cellular response to prostaglandin E stimulus | 0.009570355 |
| BP | negative regulation of intrinsic apoptotic signaling pathway in response to DNA damage by p53 class mediator | 0.009570355 |
| BP | regulation of macrophage derived foam cell differentiation | 0.009673685 |
| BP | interferon-alpha production | 0.009673685 |
| BP | interleukin-7-mediated signaling pathway | 0.009673685 |
| BP | regulation of viral entry into host cell | 0.009673685 |
| BP | myeloid leukocyte cytokine production | 0.009673685 |
| BP | negative regulation of biomineral tissue development | 0.009673685 |
| BP | regulation of reactive oxygen species metabolic process | 0.009710376 |
| BP | positive regulation of stress-activated MAPK cascade | 0.009922256 |
| BP | positive regulation of smooth muscle cell migration | 0.010242119 |
| BP | regulation of protein autophosphorylation | 0.010242119 |
| BP | reactive nitrogen species metabolic process | 0.010286194 |
| BP | temperature homeostasis | 0.010335541 |
| BP | negative regulation of establishment of protein localization | 0.010476569 |
| BP | platelet degranulation | 0.010586113 |
| BP | regulation of nitric oxide biosynthetic process | 0.010782365 |
| BP | positive regulation of erythrocyte differentiation | 0.010863713 |
| BP | positive regulation of epidermal growth factor receptor signaling pathway | 0.010863713 |
| BP | positive regulation of cytosolic calcium ion concentration involved in phospholipase C-activating G protein-coupled signaling pathway | 0.010863713 |
| BP | response to fatty acid | 0.010933235 |
| BP | regulation of interferon-beta production | 0.011174071 |
| BP | complement activation | 0.011201656 |
| BP | immune response to tumor cell | 0.011380594 |
| BP | positive regulation of cellular extravasation | 0.011380594 |
| BP | positive regulation of macrophage derived foam cell differentiation | 0.011380594 |
| BP | preganglionic parasympathetic fiber development | 0.011380594 |
| BP | regulation of interferon-gamma biosynthetic process | 0.011380594 |
| BP | regulation of single stranded viral RNA replication via double stranded DNA intermediate | 0.011380594 |
| BP | anatomical structure arrangement | 0.011380594 |
| BP | lymph node development | 0.011380594 |
| BP | regulation of killing of cells of other organism | 0.011380594 |
| BP | thymocyte apoptotic process | 0.011380594 |
| BP | regulation of glial cell migration | 0.011380594 |
| BP | extrinsic apoptotic signaling pathway | 0.011565987 |
| BP | response to antibiotic | 0.012117946 |
| BP | response to lipoprotein particle | 0.012144204 |
| BP | regulation of tumor necrosis factor secretion | 0.012144204 |
| BP | negative regulation of leukocyte migration | 0.012162191 |
| BP | actin nucleation | 0.012162191 |
| BP | regulation of glycoprotein metabolic process | 0.012162191 |
| BP | negative regulation of NF-kappaB transcription factor activity | 0.012315094 |
| BP | regulation of JUN kinase activity | 0.012315094 |
| BP | positive regulation of exocytosis | 0.012315094 |
| BP | adaptive thermogenesis | 0.012322175 |
| BP | negative regulation of endocytosis | 0.012393733 |
| BP | positive chemotaxis | 0.012393733 |
| BP | cellular response to oxidative stress | 0.012447154 |
| BP | myelination | 0.012775686 |
| BP | positive regulation of cysteine-type endopeptidase activity involved in apoptotic process | 0.012775686 |
| BP | interferon-beta production | 0.013207925 |
| BP | nucleobase-containing small molecule catabolic process | 0.013207925 |
| BP | response to interleukin-6 | 0.013207925 |
| BP | regulation of B cell apoptotic process | 0.013374454 |
| BP | I-kappaB phosphorylation | 0.013374454 |
| BP | single stranded viral RNA replication via double stranded DNA intermediate | 0.013374454 |
| BP | myeloid dendritic cell differentiation | 0.013374454 |
| BP | positive regulation of neuroinflammatory response | 0.013374454 |
| BP | negative regulation of neuroinflammatory response | 0.013374454 |
| BP | regulation of intrinsic apoptotic signaling pathway in response to DNA damage by p53 class mediator | 0.013374454 |
| BP | positive regulation of ERBB signaling pathway | 0.013517435 |
| BP | regulation of protein processing | 0.013609726 |
| BP | cellular response to peptide | 0.013850425 |
| BP | ensheathment of neurons | 0.013988939 |
| BP | axon ensheathment | 0.013988939 |
| BP | modification of morphology or physiology of other organism involved in symbiotic interaction | 0.014215887 |
| BP | positive regulation of cell-matrix adhesion | 0.014312665 |
| BP | purine-containing compound catabolic process | 0.014312665 |
| BP | G protein-coupled receptor signaling pathway, coupled to cyclic nucleotide second messenger | 0.014541986 |
| BP | regulation of protein maturation | 0.014675525 |
| BP | nucleoside catabolic process | 0.014985518 |
| BP | regulation of vascular endothelial growth factor production | 0.014985518 |
| BP | cellular response to lipoprotein particle stimulus | 0.014985518 |
| BP | response to reactive oxygen species | 0.015115706 |
| BP | positive regulation of small GTPase mediated signal transduction | 0.015116923 |
| BP | positive regulation of calcium ion transmembrane transport | 0.015116923 |
| BP | morphogenesis of an endothelium | 0.015554411 |
| BP | negative regulation of interleukin-17 production | 0.015554411 |
| BP | parasympathetic nervous system development | 0.015554411 |
| BP | endothelial tube morphogenesis | 0.015554411 |
| BP | podosome assembly | 0.015554411 |
| BP | positive regulation of protein localization to cell surface | 0.015554411 |
| BP | positive regulation of proteolysis | 0.015615367 |
| BP | positive regulation of striated muscle cell differentiation | 0.01611 |
| BP | regulation of complement activation | 0.016448398 |
| BP | regulation of type I interferon-mediated signaling pathway | 0.0165504 |
| BP | tumor necrosis factor secretion | 0.0165504 |
| BP | regulation of myoblast differentiation | 0.016704476 |
| BP | regulation of defense response to virus | 0.017147234 |
| BP | glial cell development | 0.01724581 |
| BP | regulation of protein activation cascade | 0.01724581 |
| BP | regulation of stress-activated MAPK cascade | 0.017723175 |
| BP | lymphoid progenitor cell differentiation | 0.017922251 |
| BP | platelet formation | 0.017922251 |
| BP | positive regulation of mast cell activation | 0.017922251 |
| BP | response to hydroperoxide | 0.017922251 |
| BP | negative regulation of CD4-positive, alpha-beta T cell differentiation | 0.017922251 |
| BP | regulation of monocyte differentiation | 0.017922251 |
| BP | negative regulation of T cell receptor signaling pathway | 0.017922251 |
| BP | cell differentiation involved in kidney development | 0.017994065 |
| BP | protein trimerization | 0.017994065 |
| BP | negative regulation of locomotion | 0.018074074 |
| BP | response to oxidative stress | 0.018112261 |
| BP | vascular endothelial growth factor production | 0.018213861 |
| BP | amyloid-beta clearance | 0.018213861 |
| BP | regulation of NMDA receptor activity | 0.018213861 |
| BP | positive regulation of JUN kinase activity | 0.01822948 |
| BP | negative regulation of ERK1 and ERK2 cascade | 0.01822948 |
| BP | positive regulation of response to wounding | 0.01822948 |
| BP | icosanoid biosynthetic process | 0.019347692 |
| BP | positive regulation of protein tyrosine kinase activity | 0.019347692 |
| BP | negative regulation of cytokine-mediated signaling pathway | 0.019357569 |
| BP | polyol biosynthetic process | 0.019357569 |
| BP | epidermal growth factor receptor signaling pathway | 0.019804492 |
| BP | base conversion or substitution editing | 0.020479124 |
| BP | positive regulation of phosphoprotein phosphatase activity | 0.020479124 |
| BP | platelet morphogenesis | 0.020479124 |
| BP | positive regulation of calcium ion import | 0.020479124 |
| BP | positive regulation of tumor necrosis factor secretion | 0.020479124 |
| BP | regulation of transmembrane transporter activity | 0.020751266 |
| BP | cellular response to inorganic substance | 0.020952129 |
| BP | phospholipid metabolic process | 0.020967025 |
| BP | ribonucleoside metabolic process | 0.021154068 |
| BP | nitric oxide biosynthetic process | 0.021754469 |
| BP | response to axon injury | 0.021754469 |
| BP | response to growth hormone | 0.021842813 |
| BP | transforming growth factor beta production | 0.021842813 |
| BP | Fc-epsilon receptor signaling pathway | 0.022171627 |
| BP | regulation of smooth muscle cell proliferation | 0.022171627 |
| BP | negative regulation of inflammatory response | 0.022171627 |
| BP | reactive oxygen species biosynthetic process | 0.022622576 |
| BP | regulation of apoptotic signaling pathway | 0.022725684 |
| BP | regulation of interleukin-13 production | 0.023225584 |
| BP | calcium-mediated signaling using intracellular calcium source | 0.023225584 |
| BP | positive regulation of myoblast differentiation | 0.023225584 |
| BP | immunoglobulin secretion | 0.023225584 |
| BP | cellular response to prostaglandin stimulus | 0.023225584 |
| BP | cellular response to low-density lipoprotein particle stimulus | 0.023225584 |
| BP | substrate adhesion-dependent cell spreading | 0.023343174 |
| BP | activation of JUN kinase activity | 0.023811038 |
| BP | smooth muscle cell proliferation | 0.023822798 |
| BP | response to peptide hormone | 0.023869846 |
| BP | regulation of reactive oxygen species biosynthetic process | 0.024494197 |
| BP | regulation of gliogenesis | 0.024651361 |
| BP | polyol metabolic process | 0.024651361 |
| BP | protein tetramerization | 0.024680856 |
| BP | response to calcium ion | 0.024981474 |
| BP | regulation of angiogenesis | 0.025180114 |
| BP | filopodium assembly | 0.025423961 |
| BP | protein activation cascade | 0.025594631 |
| BP | response to ethanol | 0.025712055 |
| BP | extracellular matrix disassembly | 0.025712885 |
| BP | calcium ion import | 0.025712885 |
| BP | negative regulation of cellular component movement | 0.025754108 |
| BP | axon guidance | 0.025882267 |
| BP | semaphorin-plexin signaling pathway | 0.025883311 |
| BP | response to tumor cell | 0.026161636 |
| BP | regulation of transposition | 0.026161636 |
| BP | negative regulation of transposition | 0.026161636 |
| BP | positive regulation of oligodendrocyte differentiation | 0.026161636 |
| BP | angiogenesis involved in wound healing | 0.026161636 |
| BP | negative regulation of intrinsic apoptotic signaling pathway by p53 class mediator | 0.026161636 |
| BP | phosphatidylinositol metabolic process | 0.026463316 |
| BP | neuron projection guidance | 0.02658431 |
| BP | negative regulation of response to cytokine stimulus | 0.027131976 |
| BP | G1/S transition of mitotic cell cycle | 0.0280305 |
| BP | negative regulation of toll-like receptor signaling pathway | 0.028060595 |
| BP | regulation of calcium ion import | 0.028060595 |
| BP | sensory perception of pain | 0.028179485 |
| BP | positive regulation of ion transmembrane transporter activity | 0.028179485 |
| CC | external side of plasma membrane | 2.67E-27 |
| CC | MHC protein complex | 3.37E-25 |
| CC | MHC class II protein complex | 1.72E-20 |
| CC | secretory granule membrane | 2.80E-17 |
| CC | endocytic vesicle | 2.96E-16 |
| CC | endocytic vesicle membrane | 2.88E-15 |
| CC | integral component of lumenal side of endoplasmic reticulum membrane | 7.94E-15 |
| CC | lumenal side of endoplasmic reticulum membrane | 7.94E-15 |
| CC | phagocytic vesicle | 2.57E-13 |
| CC | phagocytic cup | 2.39E-12 |
| CC | ficolin-1-rich granule | 1.24E-11 |
| CC | tertiary granule | 3.23E-11 |
| CC | ER to Golgi transport vesicle membrane | 1.14E-10 |
| CC | immunological synapse | 1.37E-10 |
| CC | clathrin-coated endocytic vesicle membrane | 1.88E-09 |
| CC | specific granule | 4.19E-09 |
| CC | membrane raft | 5.46E-09 |
| CC | COPII-coated ER to Golgi transport vesicle | 5.82E-09 |
| CC | membrane microdomain | 5.88E-09 |
| CC | tertiary granule membrane | 1.22E-08 |
| CC | membrane region | 1.39E-08 |
| CC | clathrin-coated endocytic vesicle | 1.60E-08 |
| CC | phagocytic vesicle membrane | 2.10E-08 |
| CC | specific granule membrane | 3.18E-08 |
| CC | coated vesicle membrane | 4.21E-08 |
| CC | endosome membrane | 8.30E-08 |
| CC | ficolin-1-rich granule membrane | 1.01E-07 |
| CC | clathrin-coated vesicle membrane | 1.30E-07 |
| CC | coated vesicle | 1.79E-07 |
| CC | integral component of endoplasmic reticulum membrane | 2.15E-07 |
| CC | extrinsic component of cytoplasmic side of plasma membrane | 2.23E-07 |
| CC | cytoplasmic side of plasma membrane | 3.20E-07 |
| CC | cytoplasmic vesicle lumen | 3.34E-07 |
| CC | clathrin-coated vesicle | 3.36E-07 |
| CC | vesicle lumen | 3.55E-07 |
| CC | secretory granule lumen | 4.02E-07 |
| CC | intrinsic component of endoplasmic reticulum membrane | 4.71E-07 |
| CC | actin filament | 4.71E-07 |
| CC | cytoplasmic side of membrane | 6.26E-07 |
| CC | Golgi-associated vesicle membrane | 6.69E-07 |
| CC | lysosomal membrane | 8.49E-07 |
| CC | lytic vacuole membrane | 8.98E-07 |
| CC | plasma membrane receptor complex | 3.17E-06 |
| CC | extrinsic component of plasma membrane | 3.95E-06 |
| CC | trans-Golgi network membrane | 9.56E-06 |
| CC | ficolin-1-rich granule lumen | 9.76E-06 |
| CC | extrinsic component of membrane | 1.02E-05 |
| CC | cell leading edge | 1.03E-05 |
| CC | Golgi-associated vesicle | 1.09E-05 |
| CC | vacuolar membrane | 1.54E-05 |
| CC | mast cell granule | 2.36E-05 |
| CC | podosome | 0.000155365 |
| CC | transport vesicle membrane | 0.000259238 |
| CC | early endosome membrane | 0.000287993 |
| CC | uropod | 0.000352795 |
| CC | cell trailing edge | 0.000352795 |
| CC | lamellipodium | 0.000358341 |
| CC | early endosome | 0.000370143 |
| CC | recycling endosome membrane | 0.000425089 |
| CC | recycling endosome | 0.00044154 |
| CC | transport vesicle | 0.001634496 |
| CC | endosome lumen | 0.002382073 |
| CC | protein complex involved in cell adhesion | 0.002382073 |
| CC | T cell receptor complex | 0.003019058 |
| CC | plasma lipoprotein particle | 0.003484157 |
| CC | lipoprotein particle | 0.003484157 |
| CC | early phagosome | 0.003955195 |
| CC | tertiary granule lumen | 0.004210878 |
| CC | primary lysosome | 0.004268652 |
| CC | azurophil granule | 0.004268652 |
| CC | protein-lipid complex | 0.004396697 |
| CC | late endosome | 0.005794317 |
| CC | low-density lipoprotein particle | 0.006277607 |
| CC | trans-Golgi network | 0.006788421 |
| CC | specific granule lumen | 0.007584553 |
| CC | ruffle | 0.009144717 |
| CC | vacuolar lumen | 0.009144717 |
| CC | actin cortical patch | 0.009265779 |
| CC | endocytic patch | 0.009265779 |
| CC | focal adhesion | 0.010406561 |
| CC | integrin complex | 0.010435168 |
| CC | collagen trimer | 0.010935139 |
| CC | cell-substrate adherens junction | 0.011215206 |
| CC | cell-substrate junction | 0.01237074 |
| CC | cortical actin cytoskeleton | 0.013026451 |
| CC | postsynaptic specialization, intracellular component | 0.019851168 |
| CC | PML body | 0.021005301 |
| CC | leading edge membrane | 0.021408884 |
| MF | peptide antigen binding | 6.06E-14 |
| MF | chemokine receptor binding | 4.64E-13 |
| MF | CCR chemokine receptor binding | 7.41E-13 |
| MF | cytokine binding | 3.72E-12 |
| MF | cytokine receptor activity | 5.57E-12 |
| MF | chemokine activity | 6.54E-12 |
| MF | MHC class II receptor activity | 2.51E-11 |
| MF | non-membrane spanning protein tyrosine kinase activity | 3.63E-11 |
| MF | MHC protein complex binding | 1.10E-09 |
| MF | MHC protein binding | 1.12E-09 |
| MF | GTPase regulator activity | 9.75E-09 |
| MF | antigen binding | 1.24E-08 |
| MF | G protein-coupled chemoattractant receptor activity | 3.42E-08 |
| MF | chemokine receptor activity | 3.42E-08 |
| MF | cytokine receptor binding | 3.62E-08 |
| MF | GTPase activator activity | 5.15E-08 |
| MF | MHC class I protein binding | 5.39E-08 |
| MF | SH3 domain binding | 5.93E-08 |
| MF | G protein-coupled receptor binding | 8.70E-08 |
| MF | nucleoside-triphosphatase regulator activity | 1.57E-07 |
| MF | MHC class II protein complex binding | 1.81E-07 |
| MF | cytokine activity | 1.86E-07 |
| MF | C-C chemokine receptor activity | 1.94E-07 |
| MF | C-C chemokine binding | 2.83E-07 |
| MF | protein tyrosine kinase activity | 4.97E-07 |
| MF | phosphotyrosine residue binding | 2.06E-06 |
| MF | peptide binding | 2.86E-06 |
| MF | amide binding | 3.15E-06 |
| MF | chemokine binding | 3.28E-06 |
| MF | Rho GTPase binding | 5.91E-06 |
| MF | protein phosphorylated amino acid binding | 1.72E-05 |
| MF | signaling pattern recognition receptor activity | 1.84E-05 |
| MF | carbohydrate binding | 2.19E-05 |
| MF | G protein-coupled nucleotide receptor activity | 2.43E-05 |
| MF | G protein-coupled purinergic nucleotide receptor activity | 2.43E-05 |
| MF | pattern recognition receptor activity | 2.51E-05 |
| MF | Rac GTPase binding | 3.53E-05 |
| MF | immunoglobulin binding | 5.77E-05 |
| MF | CARD domain binding | 7.64E-05 |
| MF | SH2 domain binding | 7.87E-05 |
| MF | actin binding | 0.00010127 |
| MF | phosphatase binding | 0.000136971 |
| MF | phosphoprotein binding | 0.000174294 |
| MF | phospholipid binding | 0.000208123 |
| MF | cargo receptor activity | 0.00021265 |
| MF | IgG binding | 0.000217175 |
| MF | CXCR chemokine receptor binding | 0.000217175 |
| MF | phosphatidylinositol 3-kinase binding | 0.000218852 |
| MF | purinergic nucleotide receptor activity | 0.000245808 |
| MF | nucleotide receptor activity | 0.000245808 |
| MF | coreceptor activity | 0.000293925 |
| MF | hydrolase activity, acting on carbon-nitrogen (but not peptide) bonds, in cyclic amidines | 0.000448134 |
| MF | sphingolipid binding | 0.000495517 |
| MF | amyloid-beta binding | 0.000505462 |
| MF | glycosaminoglycan binding | 0.000524955 |
| MF | signaling adaptor activity | 0.000609936 |
| MF | proteoglycan binding | 0.00061715 |
| MF | receptor ligand activity | 0.000624012 |
| MF | scavenger receptor activity | 0.000742203 |
| MF | purinergic receptor activity | 0.000744884 |
| MF | small GTPase binding | 0.000831198 |
| MF | SH3/SH2 adaptor activity | 0.000836 |
| MF | phosphatidylinositol-3,4,5-trisphosphate binding | 0.000958653 |
| MF | Ras GTPase binding | 0.001147711 |
| MF | peptidoglycan binding | 0.001358637 |
| MF | virus receptor activity | 0.001575536 |
| MF | hijacked molecular function | 0.001575536 |
| MF | guanyl-nucleotide exchange factor activity | 0.001936903 |
| MF | tumor necrosis factor receptor binding | 0.002056629 |
| MF | deaminase activity | 0.002378678 |
| MF | Rho guanyl-nucleotide exchange factor activity | 0.002397111 |
| MF | protein phosphatase binding | 0.003152002 |
| MF | death receptor activity | 0.003623982 |
| MF | profilin binding | 0.003623982 |
| MF | glucose binding | 0.003623982 |
| MF | RAGE receptor binding | 0.003623982 |
| MF | guanyl nucleotide binding | 0.003717864 |
| MF | guanyl ribonucleotide binding | 0.003717864 |
| MF | GTPase activity | 0.004025066 |
| MF | GTP binding | 0.004253444 |
| MF | G protein-coupled peptide receptor activity | 0.004341805 |
| MF | cytidine deaminase activity | 0.004725938 |
| MF | phospholipase activator activity | 0.004725938 |
| MF | purine ribonucleoside binding | 0.004798099 |
| MF | protein binding, bridging | 0.004997097 |
| MF | purine nucleoside binding | 0.005243485 |
| MF | ribonucleoside binding | 0.005399329 |
| MF | peptide receptor activity | 0.005864093 |
| MF | actin filament binding | 0.006504107 |
| MF | nucleoside binding | 0.006600665 |
| MF | phosphatidylinositol phospholipase C activity | 0.006906334 |
| MF | lipase activator activity | 0.007480641 |
| MF | cysteine-type endopeptidase regulator activity involved in apoptotic process | 0.007879483 |
| MF | Ras guanyl-nucleotide exchange factor activity | 0.00793767 |
| MF | phosphatidylinositol phosphate binding | 0.008901534 |
| MF | phospholipase C activity | 0.009026658 |
| MF | oxidoreductase activity, acting on the CH-NH2 group of donors, oxygen as acceptor | 0.011011807 |
| MF | tumor necrosis factor receptor superfamily binding | 0.011526811 |
| MF | phosphatidylinositol-3,4-bisphosphate binding | 0.011531226 |
| MF | molecular adaptor activity | 0.011797608 |
| MF | heparin binding | 0.01251369 |
| MF | NAD+ kinase activity | 0.013080344 |
| MF | Rac guanyl-nucleotide exchange factor activity | 0.013080344 |
