# Supplementary figures and images for: Overexpression of SH2D1A promotes cancer progression and is associated with immune cell infiltration in hepatocellular carcinoma via bioinformatics and in vitro study
Source: BMC Cancer. 2023 Oct 19;23:1005. doi: 10.1186/s12885-023-11315-1 (PMC10585762; doi:10.1186/s12885-023-11315-1)

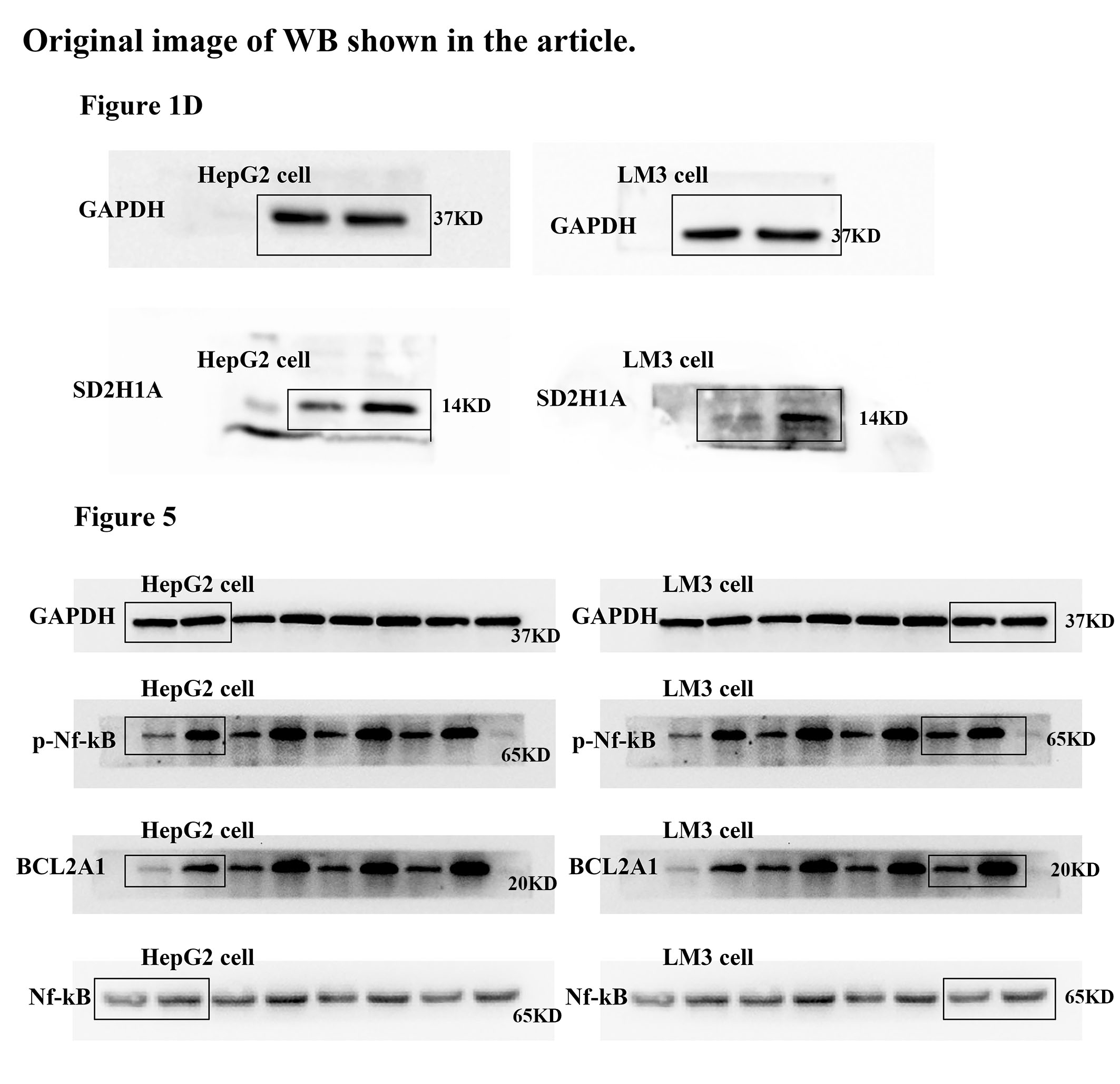

Supplement: Supplementary file 2 — Additional file 2. [file 12885_2023_11315_MOESM2_ESM.tif]
